# A Lightweight Powered Hip Exoskeleton With Parallel Actuation for Frontal and Sagittal Plane Assistance

**DOI:** 10.1109/tro.2025.3539172

**Published:** 2025-02

**Authors:** Dante Archangeli, Brendon Ortolano, Rosemarie Murray, Lukas Gabert, Tommaso Lenzi

**Affiliations:** Department of Mechanical Engineering and the Robotics Center, University of Utah, Salt Lake City, UT 84112 USA; Department of Mechanical Engineering and the Robotics Center, University of Utah, Salt Lake City, UT 84112 USA; Department of Mechanical Engineering and the Robotics Center, University of Utah, Salt Lake City, UT 84112 USA; Department of Mechanical Engineering and the Robotics Center, University of Utah, Salt Lake City, UT 84112 USA, and also with the Rocky Mountain Center for Occupational and Environmental Health, Salt Lake City, UT 84111 USA.; Department of Mechanical Engineering and the Robotics Center, University of Utah, Salt Lake City, UT 84112 USA, also with the Rocky Mountain Center for Occupational and Environmental Health, Salt Lake City, UT 84111 USA, and also with the Department of Biomedical Engineering, University of Utah, Salt Lake City, UT 84112 USA

**Keywords:** Parallel robots, physically assistive devices, prosthetics and exoskeletons, wearable robots

## Abstract

Wearable robots and powered exoskeletons may improve ambulation for millions of individuals with poor mobility. Powered exoskeletons primarily assist in the sagittal plane to improve walking efficiency and speed. However, individuals with poor mobility often have limited mediolateral balance, which requires torque generation in the frontal plane. Existing hip exoskeletons that assist in both the sagittal and frontal planes are too heavy and bulky for use in the real world. Here we present the kinematic model, mechatronic design, and benchtop and human testing of a powered hip exoskeleton with a unique parallel kinematic actuator. The exoskeleton is lightweight (5.3 kg), has a slim profile, and can generate 30 N·m and 20 N·m of torque during gait in the sagittal and frontal planes. The exoskeleton torque density is 5.7 N·m/kg—53% higher than previously possible with series kinematic design. Testing with five healthy subjects indicate that frontal plane torques applied during stance or swing can alter step width, while sagittal plane torque can assist with hip flexion and extension. A device with these characteristics may improve both gait economy and balance in the real world.

## I. Introduction

Limitations to mobility impact millions of individuals worldwide. These challenges can make simple activities like walking or climbing a flight of stairs difficult due to lack of muscle strength, coordination, or balance. Recently, powered ankle and hip exoskeletons have demonstrated the potential to improve mobility in clinical populations by generating sagittal plane torques that can support the human joint function. For example, ankle exoskeletons assisting the user’s plantar/dorsiflexion can increase level-ground self-selected walking speed in individuals poststroke [1], [2] and in children with cerebral palsy [3]. Assisting the user’s ankle plantarflexion with an ankle exoskeleton can also decrease the metabolic cost of transport during ramp and stair ascent in individuals with cerebral palsy [3], [4]. Similarly, hip exoskeletons assisting hip flexion/extension can increase level-ground self-selected walking speed in stroke survivors [5], [6], decrease metabolic cost of transport in above-knee amputees and elderly individuals [7], [8], and improve recovery after anterior and posterior slip events in elderly individuals and individuals with above knee amputation [9], [10]. Thus, walking speed and metabolic cost of transport in clinical populations can be improved through exoskeleton assistance to the sagittal plane. However, current exoskeleton assistance strategies have had limited success improving medio-lateral balance of clinical populations [9].

Medio-lateral balance is an important factor in gait. Clinical populations often struggle to maintain medio-lateral balance, putting them at high risk of falling [11], [12], [13]. To maintain mediolateral balance, healthy individuals use their hip abductor and adductor muscles to regulate hip torque in the frontal plane [14], [15]. By controlling frontal plane hip torques, healthy individuals can shift their center of mass with respect to their center of pressure when the foot is in contact with the ground. Moreover, individuals can use frontal plane hip torque to reposition their feet when the foot is off the ground, increasing their base of support in the subsequent step, which also improves balance [14], [16]. The hip abductors and adductors play a large role in both these balancing strategies, generating 0.7 to 1.0 N·m/kg of frontal plane torque during walking and climbing stairs [14], [17], [18]. Unfortunately, most people affected by mobility impairments have weakened lower limbs [19], [20], [21]. This lower limb weakness makes balance more challenging and increases the prevalence of falls [22], [23]. Therefore, powered exoskeletons could also improve balance and mobility in clinical populations by generating torques in the frontal plane.

To the best of our knowledge, only two autonomous exoskeletons have attempted to improve balance by providing assistive hip abduction and adduction torques in addition to hip flexion and extension. The first is the MindWalker, a 28 kg hip–knee–ankle exoskeleton developed for people with spinal cord injury [24]. This device provided the first demonstration of an exoskeleton that actively modifies the lateral foot placement in response to a perturbation. A 9.2 kg powered hip-only exoskeleton from North Carolina State University extended this control scheme to include admittance-based control [25]. Unfortunately, both these devices are quite heavy and bulky, which substantially reduced their usability in the real world. To address this problem, researchers developed an exoskeleton that assists the hip abduction/adduction only [26]. This device is lighter but cannot assist with body propulsion and step length symmetry, which require hip flexion/extension assistance. Thus, there is an unmet need for a lightweight and compact autonomous hip exoskeleton that can assist both hip flexion/extension and abduction/adduction.

Existing hip exoskeletons that power both sagittal and frontal plane motion are also limited by their *series* kinematic configuration. In this *series* configuration, the powered frontal plane joint meant to generate hip abduction/adduction is placed proximal to the powered sagittal plane joints meant to generate hip flexion/extension. With this configuration, at high degrees of hip flexion (e.g., during terminal swing of walking or ascending a step), exoskeleton frontal plane torques cause anatomical hip eversion/inversion torques instead of hip abduction/adduction. Therefore, powered hip exoskeletons using a *series* kinematic configuration cannot provide hip abduction/adduction torques during terminal swing in level ground walking or when climbing stairs. Moreover, in a *series* kinematic configuration, the frontal plane actuators are located in the back, preventing the exoskeleton users from sitting comfortably in a chair.

In this paper, we present a lightweight and compact powered hip exoskeleton with an alternative kinematic design that can provide hip abduction/adduction independently of the hip flexion/extension angle. Specifically, we propose a parallel kinematic actuation system that concurrently assists hip flexion/extension and abduction/adduction. Leveraging the proposed parallel actuation system, we developed a powered hip exoskeleton that is lightweight (5.3 kg), has a slim profile (adding only 3 cm posteriorly, and 8 cm laterally at the hip), and can provide high torque during gait (up to 30 N·m). This article presents the kinetostatic model and the simulation framework used to design the proposed parallel hip actuator. Benchtop testing shows the accuracy of the transmission model including feedforward friction and inertia compensations. Treadmill-based walking studies with five subjects demonstrate the ability of the proposed exoskeleton hardware and controller to provide assistance both in the sagittal and frontal planes during walking. Thus, the main contributions of this paper are the kinematic model of the actuator, the application of adaptive oscillators and phase-based torque planning to control assistive torques in the frontal plane, and the verification of the actuator and controller performance on the bench top and with human subjects. Due to its compact size, small mass, and unique kinematic configuration, we believe that the proposed powered hip exoskeleton has the potential to improve balance and reduce metabolic cost in individuals with lower-limb impairments, improving their mobility and quality of life.

## II. Modeling

### A. Powered Hip Actuator Architecture

**Fig. 1.**
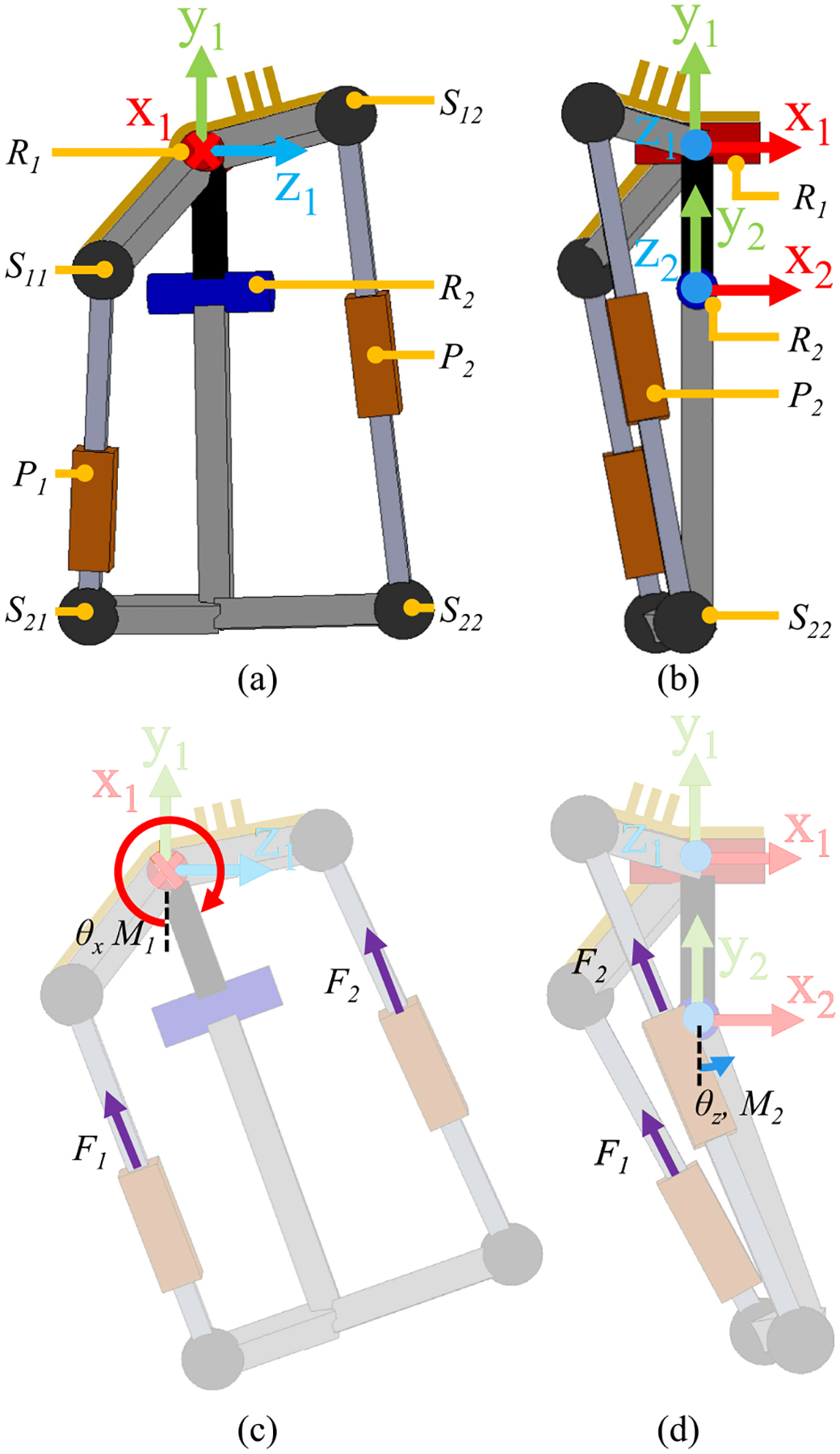
Proposed hip actuator shown from the (a) side and (b) front. The hip actuator unit is constructed from a parallel mechanism with revolute joints $R_{1}$ and $R_{2}$, spherical joints $S_{11},S_{12},S_{21}$, and $S_{22}$, and prismatic joints $P_{1}$ and $P_{2}$. When viewed from the side, the sagittal joint axis is into the page while the frontal joint axis is to the right. Hip extension and hip adduction positions and torques are considered positive. (c) Side view showing notation and direction for $\theta_{x}$ and $M_{1}$, the position and torque about the sagittal plane joint, $R_{1}$. Torque $M_{1}$ is the sum of the torques about revolute joint $R_{1}$ due to the forces $F_{1}$ and $F_{2}$. (d) Front view showing notation and direction for $\theta_{z}$ and $M_{2}$, the position and torque about the frontal plane joint, $R_{2}$. Torque $M_{2}$ is the sum of the torques about revolute joint $R_{2}$ due to the forces $F_{1}$ and $F_{2}$.

The proposed hip actuator (see Fig. 1) is based on two parallel underactuated five-bar mechanisms comprising spherical (S), revolute (R), and prismatic (P) joints. Combined, the two underactuated kinematic chains ${\text{R}}_{1}{\text{S}}_{11}{\text{P}}_{1}{\text{S}}_{21}{\text{R}}_{2}$ and ${\text{R}}_{1}{\text{R}}_{2}{\text{S}}_{22}{\text{P}}_{2}{\text{S}}_{12}$, create a fully defined kinematic system in which the linear motions of ${\text{P}}_{1}$ and ${\text{P}}_{2}$ (i.e., the linear actuators) control the rotations about ${\text{R}}_{1}$ and ${\text{R}}_{2}$ (i.e., the hip flexion/extension and abduction/adduction angles). Similar kinematic structures are used in powered prosthetic ankles and humanoid robots [27], [28], [29].

### B. Kinetostatic Model

The parallel hip actuator provides hip extension/flexion and hip adduction/abduction torques [see Fig. 1(c) and (d), respectively]about revolute joints $R_{1}$ and $R_{2}$, respectively. These torques, labeled $M_{1x}$ and $M_{2z}$, are defined by the forces $F_{1}$ and $F_{2}$ produced by two linear actuators, modeled as prismatic joints $P_{1}$ and $P_{2}$. The relationship between these forces and torques can be expressed as the inverse of the transpose of the velocity Jacobian J

(1) $$\left[\begin{array}{l} M_{1x}\\ M_{2z} \end{array}\right]=J^{-T}\left[\begin{array}{l} F_{1}\\ F_{2} \end{array}\right].$$

Moreover, the velocity Jacobian relates the output joint velocities of $R_{1}$ and $R_{2}$ (${\dot{\theta }}_{x}$ and ${\dot{\theta }}_{z}$, respectively) to the velocities of linear actuator $P_{1}$ and $P_{2}$

(2) $$\left[\begin{array}{c} {\dot{\theta }}_{x}\\ {\dot{\theta }}_{z} \end{array}\right]=J\left[\begin{array}{l} {\dot{P}}_{1}\\ {\dot{P}}_{2} \end{array}\right].$$

Following the definition of (1), the components of $J^{-T}$ are ratios between the applied force of the linear actuators and their respective moments. We define the inverse of the transpose of the velocity Jacobian as follows:

(3) $$J^{-T}=\left[\begin{array}{ll} {\text{TR}}_{11x} & {\text{TR}}_{12x}\\ {\text{TR}}_{21z} & {\text{TR}}_{22z} \end{array}\right]$$

where ${\text{TR}}_{11x}$ is the ratio between the applied force $F_{1}$ and its corresponding moment about and in the direction of joint $R_{1}\left(M_{11x}\right).$ ${\text{TR}}_{12x}$ is the ratio between the applied force $F_{2}$ and its corresponding moment about and in the direction of joint $R_{1}\left(M_{12x}\right)$. Similarly, ${\text{TR}}_{21z}$ and ${\text{TR}}_{22z}$ are the ratios between the applied forces $F_{1}$ and $F_{2}$ and their respective moments about and in the direction of $R_{2}$ ($M_{21z}$ and $M_{22z}$).

**Fig. 2.**
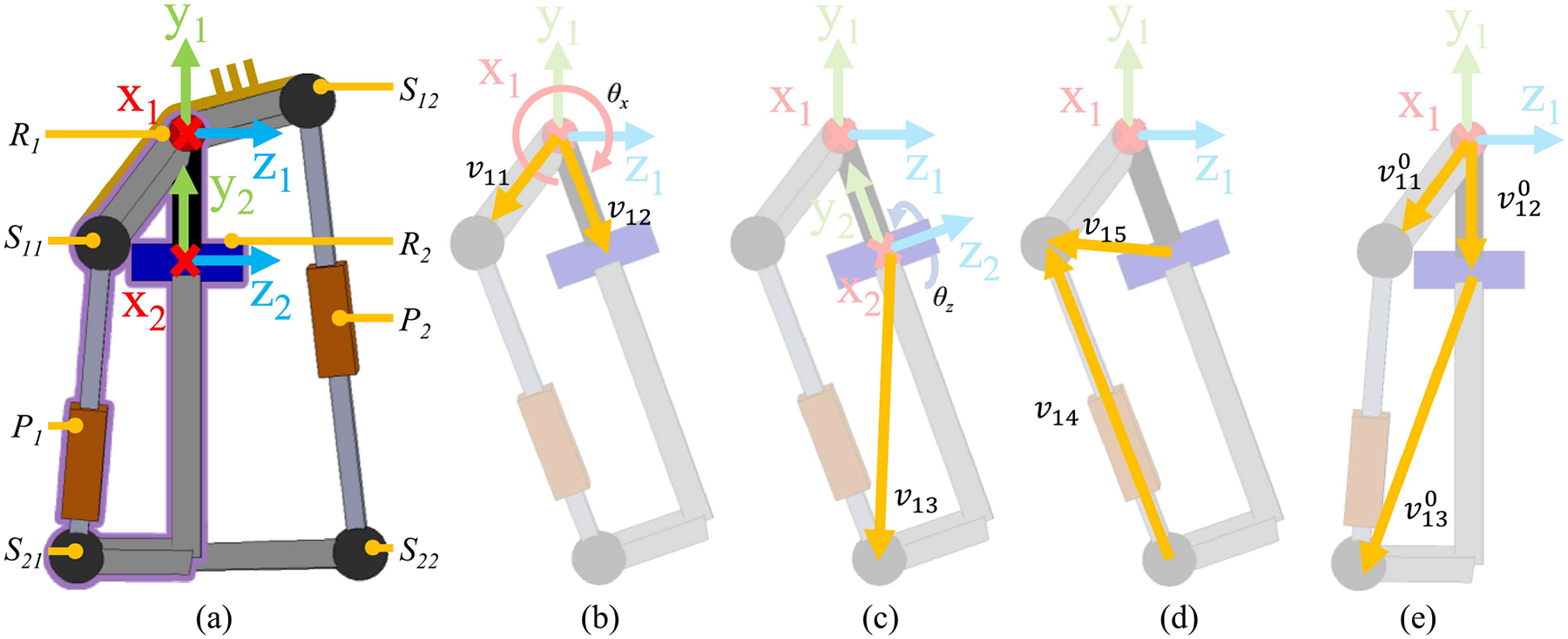
(a) Side view of parallel hip actuator with one kinematic chain outlined in purple. The single kinematic chain is used to calculate the ratio of the applied force to resultant joint torque. The single kinematic chain articulated to 20° flexion with (b) vectors $v_{11}$ and $v_{12}$, and joint angle $\theta_{x}$, (c) vectors $v_{13}$ and joint angle $\theta_{z}$, and (d) vectors $v_{14}$ and $v_{15}$. (e) Kinematic chain in the zero-angle configuration.

The specific ratios between the applied forces (i.e., $F_{1}$ and $F_{2}$) and the resultant moments ($M_{1x}$ and $M_{2z}$) can be found by independently solving the kinematic chains $R_{1}S_{11}P_{1}S_{21}R_{2}$ and $R_{1}R_{2}S_{22}P_{2}S_{12}$ with grounded components $R_{1},S_{11}$, and $S_{12}$ (see Figs. 1 and 2). Here we will solve kinematic chains $R_{1}S_{11}P_{1}S_{21}R_{2}$ to determine the relationship between $M_{11x}$ and $M_{21z}$ and $F_{1}$.

The kinematic chains $R_{1}S_{11}P_{1}S_{21}R_{2}$ can be described by the dimensions of link $R_{1}S_{11},R_{1}R_{2}$, and $R_{2}S_{21}$ and the joint angles $\theta_{x}$ and $\theta_{z}$ (see Figs. 1 and 2). These links are modeled as vectors $v_{11},v_{12}$, and $v_{13}$, respectively. Vector $v_{11}$ is grounded [see Fig. 2(b)]. Vector $v_{12}$ rotates about the $x_{1}$ axis by $\theta_{x}$ [see Fig. 2(b)]. Vector $v_{13}$ is fixed to the distal end $v_{12}$ and rotates about $z_{2}$ by $\theta_{z}$ [see Fig. 2(c)]. The specific dimensions of each vector with respect to coordinate system 1 when $\theta_{x}$ and $\theta_{z}$ are both zero [see Fig. 2(e)] are listed in Table I. These vectors are labeled with a superscript 0 as in $v_{11}^{0}$ to distinguish them from their respective value after a rotational transform is applied. The vectors are described as follows:

(4) $$v_{11}=v_{11}^{0}\;v_{11}^{0}=\left[\begin{array}{l} x_{11}\\ y_{11}\\ z_{11} \end{array}\right]$$

(5) $$[-2pt]v_{12}=R_{x}\left(\theta_{x}\right)v_{12}^{0},\;v_{12}^{0}=\left[\begin{array}{l} x_{2}\\ y_{2}\\ z_{2} \end{array}\right]$$

(6) $$v_{13}=R_{x}\left(\theta_{x}\right)R_{z}\left(\theta_{z}\right)v_{13}^{0},\;v_{13}^{0}=\left[\begin{array}{l} x_{12}\\ y_{12}\\ z_{12} \end{array}\right]$$

where $R_{x}\left(\theta_{x}\right)$ and $R_{z}\left(\theta_{z}\right)$ are standard three-dimension rotation matrixes about the x and z axes, respectively.

We construct vectors $v_{14}$ and $v_{15}$ between $S_{11}$ and $R_{2}$, and $S_{11}$ and ${\text{S}}_{21}$ from $v_{11},v_{12}$, and $v_{13}$ [see Fig. 2(d)].


(7) $$v_{14}=v_{11}-v_{12}-v_{13}$$

(8) $$v_{15}=v_{11}-v_{12.}$$

Using these vectors, we calculate the transmission ratio relating the force $F_{1}$ applied by prismatic joint $P_{1}$ to the torques $M_{11x}$ and $M_{21z}$

(9) $${\text{TR}}_{11x}=-x\cdot \frac{v_{11}\times v_{14}}{\left|v_{14}\right|}$$

(10) $${\text{TR}}_{21z}=-R\left(\theta_{x}\right)z\cdot \frac{v_{15}\times v_{14}}{\left|v_{14}\right|}.$$

A similar model is constructed from $R_{1}R_{2}S_{22}P_{2}S_{12}$ to calculate the transmission ratio between $M_{12x}$ and $M_{22z}$ and $F_{2}$ applied by prismatic joint $P_{2}$ using the shared joints and vector $R_{1},R_{2}$, and $v_{12}$, and independent vectors $v_{21}$ and $v_{23}$ constructed from design parameters $x_{21},x_{22},y_{21},y_{22},z_{21},z_{22}$ (see Table I).

Force $F_{1}$ and $F_{2}$ are generated by linear actuators powered by high performance brushless motors (see Figs. 1 and 4). These actuators are built with a primary gear stage and a ball screw with transmission ratios ${\text{TR}}_{g}$ and ${\text{TR}}_{bs}$, respectively. Equations (1) and (2) can be modified to relate the joint torques $M_{1x}$ and $M_{2z}$ and the joint velocity ${\dot{\theta }}_{x}$ and ${\dot{\theta }}_{z}$ to the motor torques ($\tau_{m1}$ and $\tau_{m2}$) and velocities (${\dot{\theta }}_{m1}$ and ${\dot{\theta }}_{m2}$)

(11) $$\left[\begin{array}{l} \tau_{m1,\text{static}}\\ \tau_{m2,\text{static}} \end{array}\right]=J^{T}\left[\begin{array}{c} M_{1x}\\ M_{2z} \end{array}\right]\left(\frac{1}{{\text{TR}}_{g}{\text{TR}}_{bs}}\right)$$

(12) $$\begin{array}{cc} \left[\begin{array}{l} {\dot{\theta }}_{m1}\\ {\dot{\theta }}_{m2} \end{array}\right] & =J^{-1}\left[\begin{array}{c} {\dot{\theta }}_{x}\\ {\dot{\theta }}_{z} \end{array}\right]\left({\text{TR}}_{g}{\text{TR}}_{bs}\right). \end{array}$$

Thus, using (9)–(12) it is possible to relate hip actuator joint torques and velocities to motor torques and velocities. These relationships are used in a simulation framework to identify critical exoskeleton dimensions.

### C. Simulation Framework

Similar to our previous work [30], [31], [32], [33], [34], we use a simulation framework to guide the design of the powered hip actuator. The simulation framework captures the dynamic behavior of the proposed parallel actuator mechanism by integrating an electromechanical model of the brushless motor with the kinetostatic model shown in the previous section. The simulation framework takes as input the desired torque, position, and velocity of the output hip abduction and hip extension joints derived from walking [17] and stair climbing [18] datasets. Based on these inputs, the framework calculates motor current and voltage for a specific parameter set describing the dimensions of the linkages.

As is commonly done in the field [35], [36], the dynamic model accounts for the inertial torque due to the motor $\left(H_{m}\right)$ and the transmission system $\left(H_{\text{TR}}\right)$. We also account for mechanical losses of the linear actuator using an efficiency term $\eta_{\text{TR}}$. Using the motor torque (11) and velocity (12) we calculate the motor current ($i_{m1}$ and $i_{m2}$) and subsequently motor voltage ($V_{m1}$ and $V_{m2}$) for each actuator as follows:

(13) $$\left[\begin{array}{l} i_{m1}\\ i_{m2} \end{array}\right]=\frac{1}{k_{t}}\left(\frac{1}{\eta_{TR}}\left[\begin{array}{c} \tau_{m1,\text{static}}\\ t_{m2,\text{static}} \end{array}\right]+\left(H_{m}+\frac{H_{TR}}{TR_{g}^{2}}\right)\left[\begin{array}{l} {\ddot{\theta }}_{m1}\\ {\ddot{\theta }}_{m2} \end{array}\right]\right)$$

(14) $$\left[\begin{array}{l} V_{m1}\\ V_{m2} \end{array}\right]=R_{m}\left[\begin{array}{l} i_{m1}\\ i_{m2} \end{array}\right]+k_{t}\left[\begin{array}{l} {\ddot{\theta }}_{m1}\\ {\ddot{\theta }}_{m2} \end{array}\right]$$

where $R_{m}$ is the resistance of the motor windings and $k_{t}$ is the motor constant. The effect of inductance is neglected in this model. After the motor voltage and current are calculated, the simulation framework checks that the motor root mean square current is less than the nominal motor current ($i_{\text{nom}}$) and that the motor voltage is less than the supply voltage $\left(V_{s}\right)$, accounting for losses in the motor driver ($\eta_{\text{driver}}$):

(15) $$\left(\left[\begin{array}{c} i_{m1}^{\text{rms}}\\ i_{m2}^{\text{rss}} \end{array}\right]<i_{\text{nom}}\right)\left(\left[\begin{array}{l} \left|V_{m1}\right|\\ \left|V_{m2}\right| \end{array}\right]<\eta_{\text{driver}}V_{s}\right).$$

Using the simulation framework, we can explore the design space to understand the effect of the different parameters on performance.

## III. Design

### A. Simulations

We use an iterative design approach to explore the influence of critical design parameters on the powered hip actuator performance and size. The simulation framework takes as input, a list of powered hip design parameters, human hip biomechanics for walking [17] and stair ascent [18], and motor specifications. Leveraging the kinetostatic model and simulation framework, we predict motor performance. Table I shows the selected design parameters. The large number of design parameters and preference for a search grid with a small step size creates a simulation with high time complexity. The design space can be reduced by constraining the shape of the hip actuator and identifying parameters with reduced impact on the hip actuator performance.

**Fig. 3.**
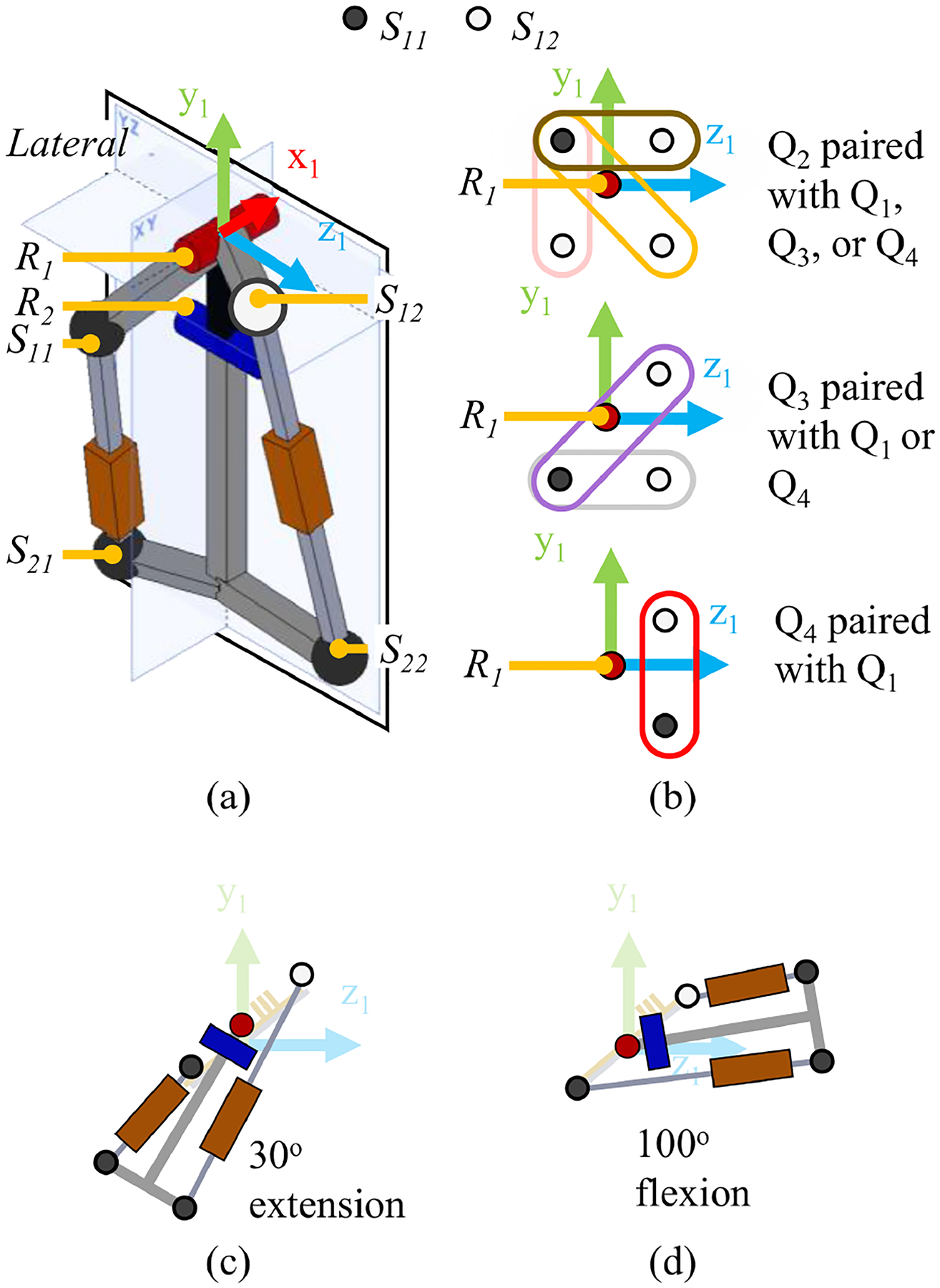
(a) Isometric view of schematic design. The lateral side of the YZ plane for a right-sided actuator is called out. (b) Six potential locations for the actuator primary spherical joints $S_{11}$ and $S_{12}$. (c), (d) Actuator pivots must be located in the 1 st and 3 rd quadrants to create a compact design and to prevent collisions between the two linear actuators, frame, and crank.

The proposed hip actuator can be built such that the proximal spherical joints of the linear actuators [see $S_{11}$ and $S_{12}$, Fig. 3(a)] are located in one of the eight cartesian octants. To reduce the actuator lateral size, the proximal spherical joints should be placed on the same side of the YZ plane and close to $R_{1}$. To reduce potential contact with the user, the linear actuators and thus spherical joints should be placed on the lateral aspect of YZ plane. When designing a right-side hip actuator, this would force the x coordinate of $v_{11}$ and $v_{21}$ to be negative which reduces the 8 octants to four quadrants in the YZ plane [see Fig. 3(a)]. The proximal spherical joints can be located in the same quadrant but, due to the size of the spherical joint (e.g., about 25 mm in diameter), this would be difficult to manufacture while still maintaining a compact actuator. Based on these design constraints, there are six general combinations for the location of the spherical joints [i.e., $S_{11}$ located in quadrant 2 and $S_{12}$ located in quadrant 1, 3, or 4; $S_{11}$ located in quadrant 3 and $S_{12}$ located in quadrant 1 or 4; $S_{11}$ located in quadrant 4 and $S_{12}$ located in quadrant 1; Fig. 3(b)]. Of the six combinations, only one combination [$S_{11}$ located in quadrant 3 and $S_{12}$ located in quadrant 1, Fig. 3(c) and (d)] allows for 30° of hip extension and 100° of hip flexion while placing the proximal spherical joints close to $R_{1}$. Placement of the distal spherical joints ($S_{21}$ and $S_{22}$) has little impact on the device performance and was selected to increase the minimum distance between the two linear actuators across the device range of motion. Using these restrictions, the number of simulation parameters can be reduced.

In addition to restricting the domain of design parameters that describe the kinematic behavior of the device, it is also possible to predetermine a finite number of ball screw and primary gear stage transmission ratios (${\text{TR}}_{bs},{\text{TR}}_{g}$ respectively). Notably, these mechanical elements scale the velocity Jacobian as in (11) and (12). Thus, for the same peak transmission ratio, decreases in the velocity Jacobian can be compensated by increases in the ball screw and primary gear stage transmission ratio. To satisfy our desire for a compact device, the spherical joints are placed close to $R_{1}$ which sets the peak transmission ratio achieved across the range of motion. In doing so, we can also identify combinations of stock ball screw and gear pairs that scale the transmission ratio to an acceptable range. Thus, the primary gear ratio and ball screw ratio can be reduced to a small number of combinations based on the range of acceptable proximal spherical joint geometries and the availability of stock components.

During level ground walking, the simulation framework predicts that the proposed hip actuator built according to design parameters in Table I can produce 28.5 N·m of frontal-plane assistance and 25.5 N·m of sagittal-plane assistance with a root mean square current and peak voltage of 4.64 A and 14.4 V, respectively. These peak torque values correspond to 28.2% of the biological torques for a 95th percentile male (i.e., 122.6 kg) [17], [37]. At this level of assistance, the hip actuator injects a total of 10.9 J of mechanical energy into the gait cycle and absorbs a total of 7.2 J of mechanical energy. Accounting for thermal losses, the actuator consumes 24.5 J of electrical energy per stride. The predicted maximum torque increases when the exoskeleton acts to produce torque only in the frontal or sagittal plane. Specifically, the simulations predict that the actuators can produce 30.3 and 48.2 N·m when assisting in only the sagittal or frontal plane, respectively.

During stair ascent, the simulation framework predicts that the proposed hip actuator can produce 28.4% of the biological torque for a 95th percentile male [18], [37]. These values correspond to 34.0 N·m of frontal plane assistance and 14.9 N·m of sagittal plane assistance, with a root mean square current and peak voltage of 4.64 A and 12.9 V, respectively. At this level of assistance, the hip actuator injects a total of 16.6 J of mechanical energy into the gait cycle and absorbs a total of 1.1 J of mechanical energy. Accounting for thermal losses, the actuator consumes 38.1 J of electrical energy per stride. When acting to produce only frontal plane torque or only sagittal plane torque, the actuators can produce up to 38.4 and 67 N·m of assistance, which is substantially higher than when producing torque in both planes simultaneously. Thus, the simulation framework predicts that the proposed parallel hip actuator can provide similar performance to existing autonomous exoskeletons [25], [38].

### B. Mechanics

**Fig. 4.**
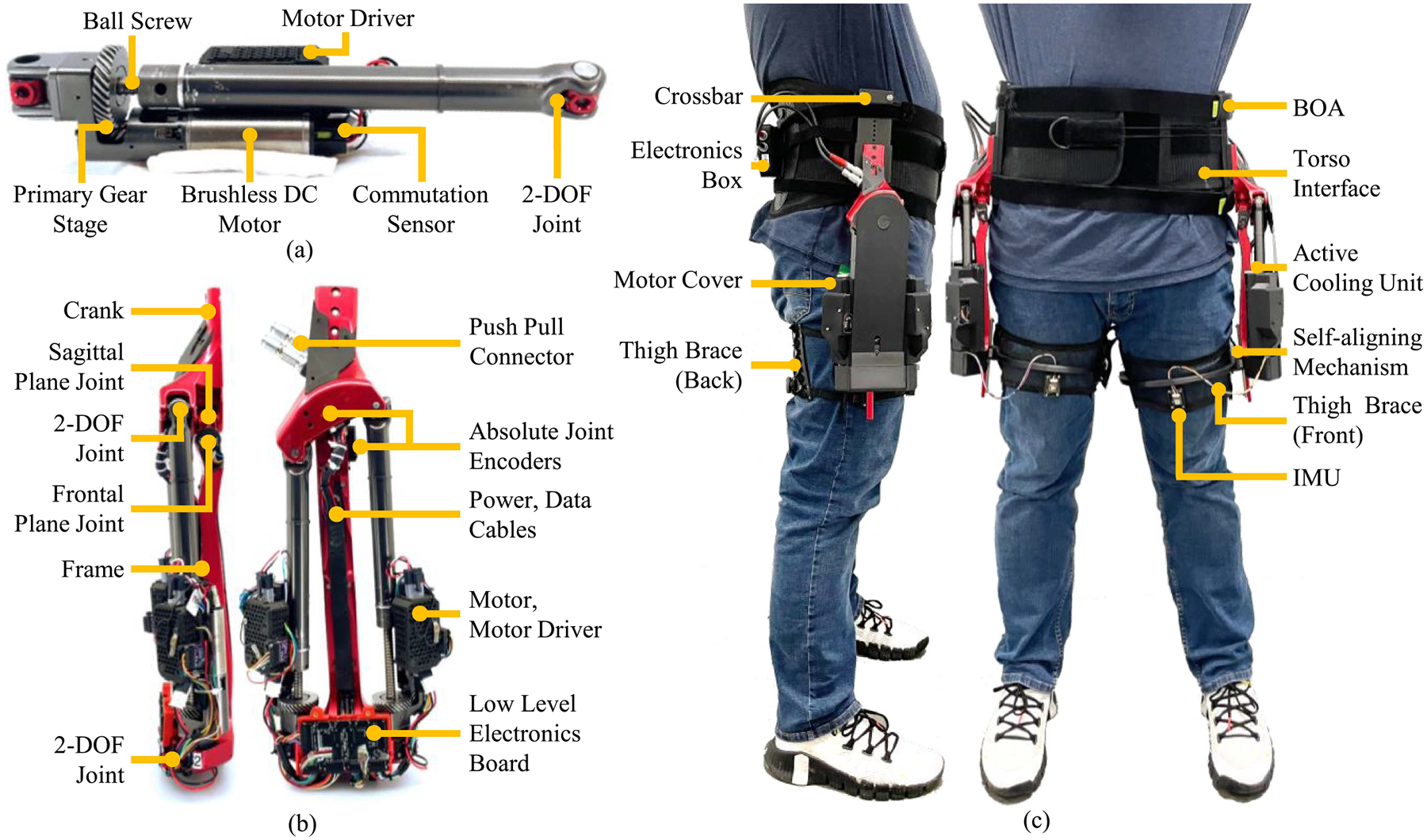
(a) Linear actuator is built from a brushless dc motor, helical gear stage, and a ball screw assembly. A motor control unit is mounted directly to the linear actuator to reduce electrical noise. A protective cover (not pictured) reduces actuator noise, minimizes debris penetration, and actively dissipates motor heat. (b) Two linear actuators are connected to the powered hip actuator crank and frame through 2-DOF joints. A low-level electronic board reads sensor data from the absolute joint encoders and the inertial measurement unit, and communicates with the motor controller. (c) Realized exoskeleton on a subject. The hip actuator is connected to the subject’s pelvis through a rigid crossbar and pelvis wrap and connected to the user’s thigh through a rigid thigh brace and flexible cuff. Both the pelvis wrap and thigh cuff can be tightened with a BOA closure system. The high-level electronics and battery are placed posteriorly and secured to the pelvis wrap.

The powered hip actuator is built based on the design parameters reported in Table I. Each hip actuator is powered by two identical linear actuators [see Fig. 4(a)]. Each linear actuator comprises a brushless DC motor (Maxon, 323218, EC-4pole 22 mm, 90 W, 24 V), a primary gear stage (Boston Gears, 3:1), and a ball screw (Ewellix, 8 × 2.5 mm). The primary gear stage and ball screw are covered by plastic shields that protect the transmission from debris. An active cooling system provides forced heat convection, reducing the motor thermal resistance. A similar system has demonstrated a 39% increase in the continuous current of the motor [30]. The linear actuators are connected to the hip actuator frame and crank through passive two-degree of freedom joints [see Fig. 4(a) and (b)]. These joints construct a 2-force body about the linear actuator and accommodate frontal and sagittal plane hip motion. The powered hip frame, crank, and two linear actuators construct two coupled five-bar mechanisms. Independently, these mechanisms are similar to a four-bar inverted slider crank. The powered hip frame, crank, connecting bar, and two-degree of freedom joints are made from custom-machined aluminum. Dry bushings (IGUS) provide low friction and low-weight revolute motion.

The powered hip crank is rigidly connected to a compliant torso interface [see Fig. 4(c)]. Similar to our previous work [39], the torso interface is built from a compliant lower spine orthosis (Ottobock). Compliant thermoplastic pads are placed beneath the orthosis. The exoskeleton crank is connected to a rigid crossbar that is mounted on the thermoplastic pads. The battery and high-level electronic motherboard are placed posteriorly on the torso interface. The compliant torso interface can be adjusted to fit both small and large individuals and the hip actuator can be mounted at various points along the rigid crossbar.

The frame of the powered hip actuator is connected to a thigh brace through a self-aligning mechanism [see Fig. 4(b) and (c)]. The mechanism is built from a prismatic and revolute joint which allows for dynamic alignment of the powered joints to the human hip joint center of rotation [40], thus reducing spurious forces and torques between the exoskeleton and the user [41], [42]. The thigh brace is made from a rigid bar and mesh band. The rigid bar is located on the anterior aspect of the thigh and connected to the mesh band at the medial and lateral aspects. The posterior aspect of the mesh band can be tightened around the thigh using a BOA lacing system (Click-Medical). This system distributes the forces of the exoskeleton equally across the brace [42], [43].

### C. Embedded Sensing and Power Electronics

**Fig. 5.**
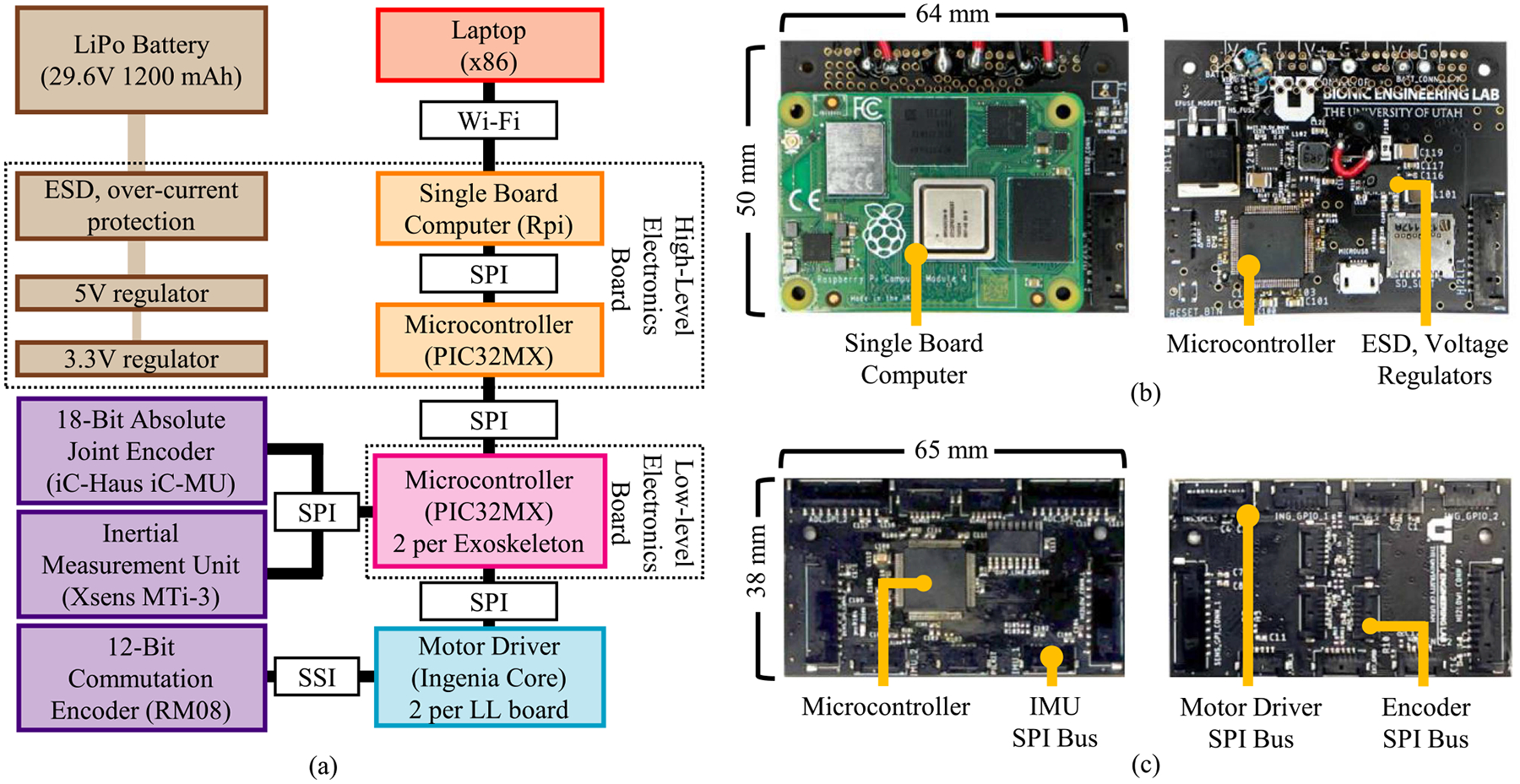
(a) Schematic architecture of electrical system, and (b) realization of high-level (HL) and (c) low-level (LL) electronic boards. A high-level board streams data to a laptop computer with GUI interface. Through the GUI, control parameters of the exoskeleton can be updated. The high-level board coordinates communication between low-level boards located in both the right and left powered hip actuators. The low-level boards collect sensor information, run low-level control routines, and communicate with the motor drivers.

The embedded electronic system consists of a high-level motherboard, two low-level boards, four motor drivers, and a suite of digital sensors (see Fig. 5), which consume an estimated 6 W of electric power in their idle state. A 1200 mAh 8-cell lithium polymer battery (128 kJ) powers the exoskeleton. Based on the hip actuator expected electrical energy consumption of 24.5 J per stride, we estimate that the bilateral exoskeleton can provide the maximum assistive torque during walking for more than 4600 steps, which is sufficient to perform laboratory research studies.

The high-level motherboard performs limited control routines, wireless and wired data communication, and power management. A single-board computer (Raspberry Pi Compute Module 4) computes desired joint torques and streams data wirelessly to a host computer which runs a graphical user interface (GUI). Using the GUI, the experimenter can monitor exoskeleton data online and modify all the high-level control parameters while the device is operating. A microcontroller (PIC32) performs communication and data sharing between the single-board computer and the low-level boards. Power management ICs protect against electro-static discharge, inrush current, overcurrent, and low voltage, and scale the supply voltage to 5 and 3.3 V to power the single-board computer, microcontrollers, motor controller logic, and sensors. The high-level electronics board is located on the exoskeleton pelvis interface.

Each low-level electronics board performs data collection and time-critical control routines. A microcontroller (PIC32) communicates over SPI with physical sensors, two motor drivers, and the high-level board. The physical sensors include 18-bit absolute joint encoders (iC Haus iC-MU with 16 Pole, 1.28 pitch Nonius encoder) that read the exoskeleton frontal and sagittal plane joint angle and an inertial measurement unit (XSENS MTi-3) that provides exoskeleton frame acceleration, orientation, and angular velocity. The motor controllers (Ingenia Capitan Core) conduct motor commutation using a 12-bit absolute encoder (RLS RM08) and run closed-loop field-oriented current control at 100 kHz. One low-level board is located on each exoskeleton frame and one motor-driver and commutation sensor are mounted to each linear actuator.

A dedicated high-power cable supplies electrical power to each motor controller while a dedicated data cable provides low-level power and communication between the high and low-level electrical boards. Push–pull connectors (LEMO, B Series) provide secure connections for external cables.

### D. Weight Breakdown

The weight of individual exoskeleton components is shown in Table II. The assembled bilateral exoskeleton weighs 5.3 kg. Each hip actuator weighs 1.7 kg or 32.4% of the exoskeleton mass, with the majority of mass located in the crank, exoskeleton frame, and connecting bar (736 g). Each linear actuator weighs 406 g. The low-level electronics board, sensors, and power and data cables within the exoskeleton weigh 71 g. The orthosis and braces weigh a total of 1.3 kg (24.5% of the exoskeleton mass). The pelvis orthosis weighs 942 g, while the thigh orthosis and passive degree of freedom weigh 178 g per side. The high-level electronics unit, battery, and power and data cables weigh 603 g (11.4% of the exoskeleton mass).

## IV. Control

**Fig. 6.**
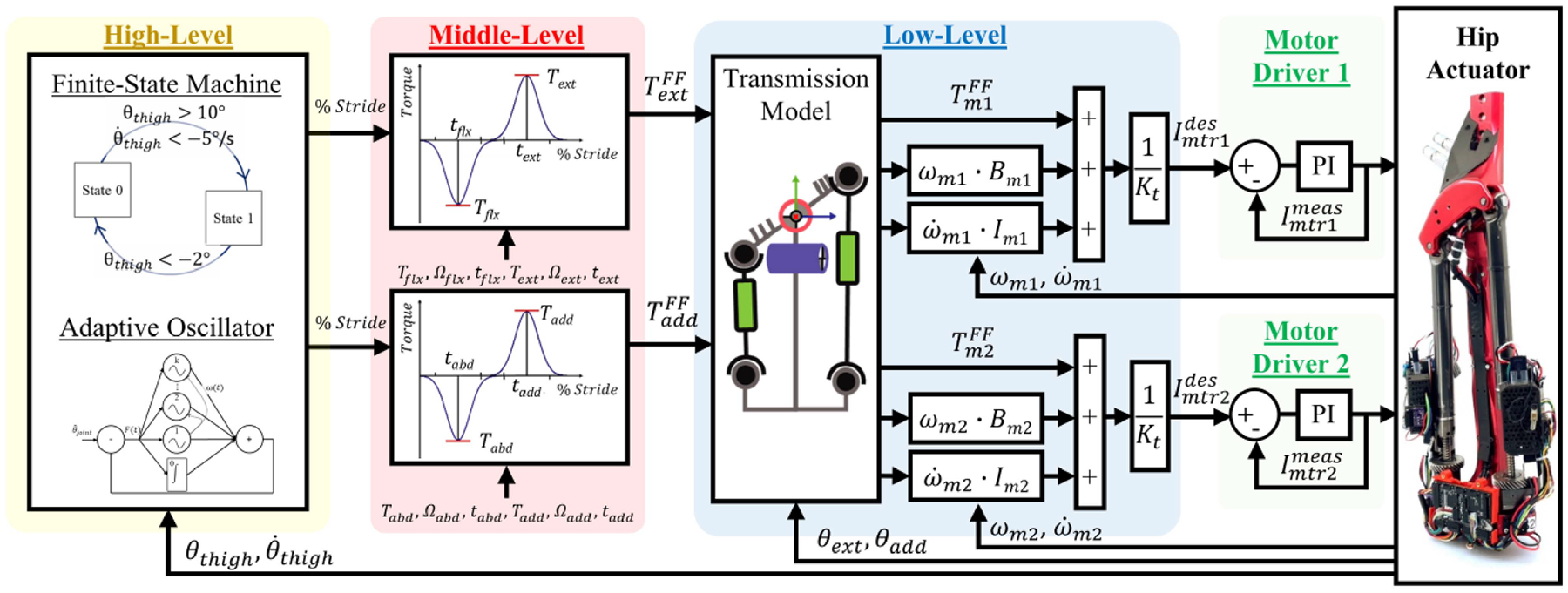
Control diagram for the powered hip actuator. At the high level, an adaptive oscillator provides a noise and delay free estimate of sagittal plane thigh orientation and stride frequency. In conjunction with a finite-state machine, these tools estimate percent stride completion. A torque planner at the mid-level generates assistive torque profiles based on stride estimate and experimenter tunable parameters. The feed-forward frontal and sagittal plane torques are transformed into motor torques based on the transmission model. These torques are combined with feed-forward inertia and friction compensations. The total feed-forward motor torque is divided by the motor torque constant to obtain the desired motor current. The motor drivers perform closed-loop current control to drive the linear actuators of the powered hip actuator.

The exoskeleton uses a hierarchical control structure similar to previous work [30] (see Fig. 6). The high-level controller generates a real-time gait phase estimate. The gait phase estimate is the product of a gait timer and a gait cadence estimate generated by an adaptive oscillator [44], [45]. The gait timer is reset every cycle by a state machine that identifies peak hip flexion. The parameters of the state machine can be selected to be robust across ambulation tasks [30]. The phase estimate generated by the high-level controller is utilized by a mid-level torque planner. The torque planner generates desired assistive torque profiles for both the sagittal and frontal plane exoskeleton joints. Each assistive torque profile is the sum of two Gaussian functions in the gait-phase domain. In the sagittal plane, this assistive strategy has successfully reduced the metabolic cost of walking of individuals with lower limb amputation [7]. A similar approach generating abduction assistance during stance has reduced the metabolic cost of walking for healthy individuals [46].

The duration, magnitude, and phase-timing of the Gaussian functions is manually tuned to achieve the desired torque profiles. The desired joint torques are converted by the low-level controller into desired motor currents for the linear actuator modules. Corrections are made for inefficiencies in the hip exoskeleton actuation. Additional feed-forward friction and inertia terms are added to the desired current to compensate for the linear actuator dynamics. The desired motor currents are sent to motor drivers which perform closed-loop current control and torque vectoring.

The high and low-level controllers (see Fig. 6) run on the microcontroller on the low-level electronics board at 1000 Hz [see Fig. 5(a) and (c)], while the mid-level torque planner (see Fig. 6) runs on the single-board computer on the high-level electronics board at 500 Hz [see Fig. 5(a) and (b)].

## V. Benchtop Testing

**Fig. 7.**
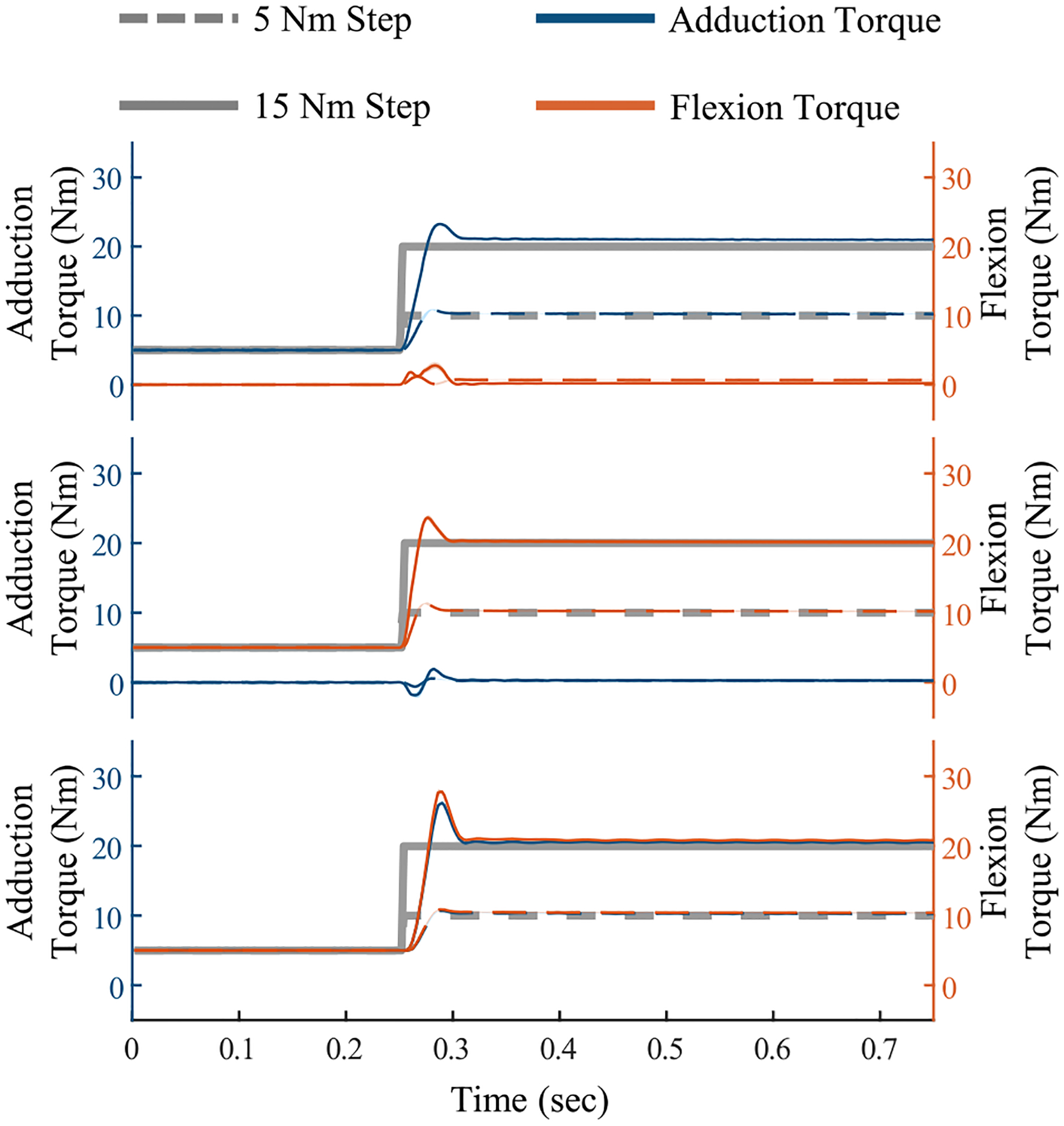
Exoskeleton generated torque in response to step increase in desired torque. In all conditions, 5 N·m of preload torque were applied before administering the step increase in torque. Frontal plane torque (adduction) is shown in blue while sagittal plane torque (flexion) is shown in orange. Two steps are applied, one of 5 N·m and one of 15 N·m. Exoskeleton performance in response to a step increase in (top) frontal plane torque, (middle) sagittal plane torque, and (bottom) both frontal and sagittal plane torque.

We estimated the hip actuator steady-state error, actuator bandwidth, and impedance by performing benchtop tests. Similar to our prior work [30], we connected the hip actuator crank to a grounded six-axis force-torque sensor (Sunrise Instruments M3713D). The hip actuator joints were both set to zero degrees and the distal portion of the exoskeleton frame was also grounded. Five N·m of preload was applied, and we commanded torque steps of 5 or 15 N·m in three separate conditions: frontal plane torque, sagittal plane torque, and combined torque. Forces and torques measured at the load cell were reflected to the hip actuator joint centers. Each step was conducted 5 times, and the results are reported in (see Fig. 7). The rise time, percent overshoot, and steady-state error, were extracted from each trial and averaged (see Table III). Across the 6 experimental conditions, the risetime, percent overshoot, and rms steady state error are between 9.4 and 17.2 ms, 6.9% and 39.3%, and 0.2 and 1.0 N·m.

**Fig. 8.**
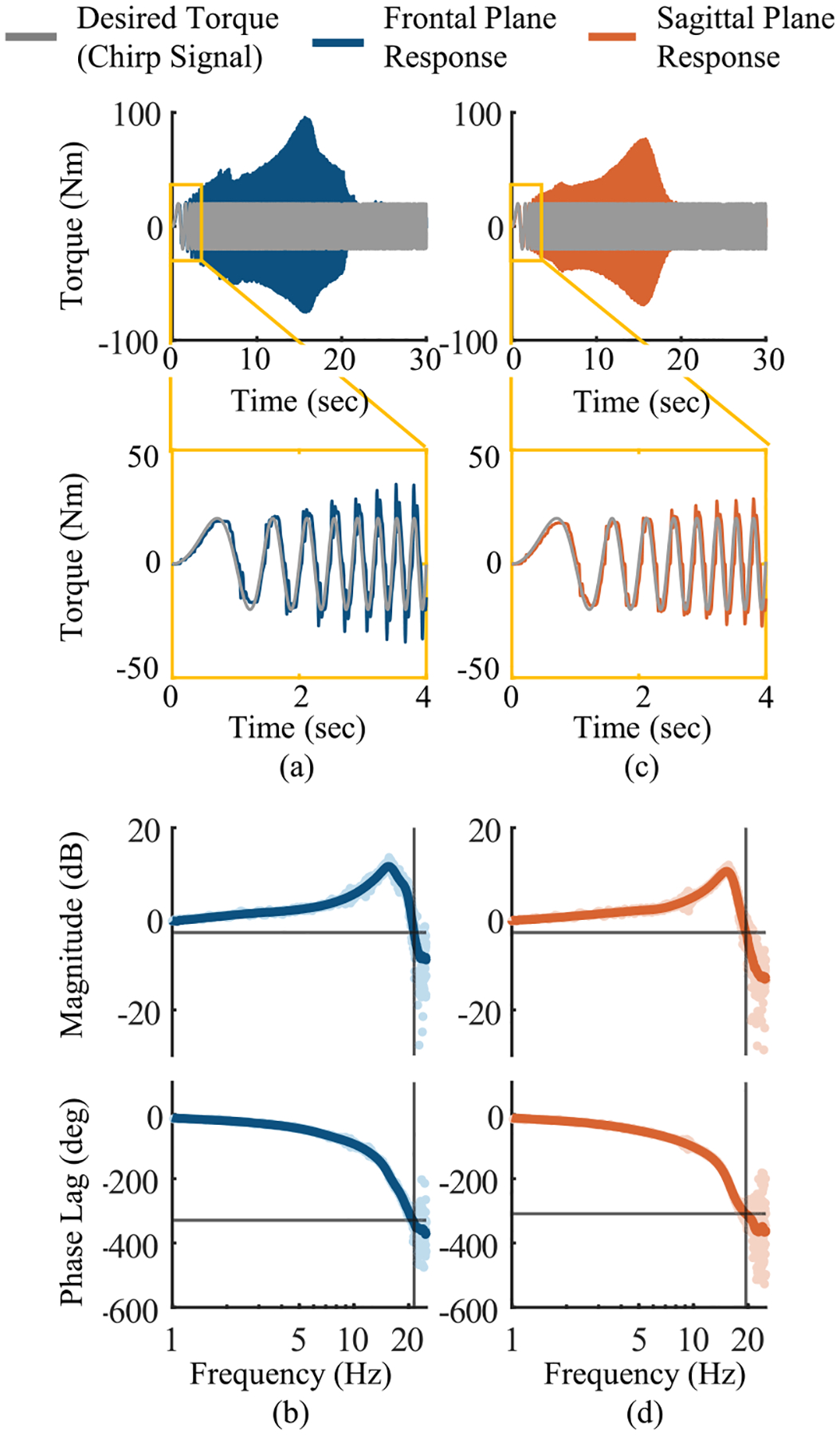
Estimate of the feed-forward bandwidth of the hip actuator in the (a), (b) frontal plane and (c), (d) sagittal plane. (a) Desired torque in the frontal plane was set to follow a sinusoidal profile with magnitude of 20 N·m while the Sagittal plane torque was set to zero. The period corresponding to 0 to 4 Hz is shown in close-up. (b) desired and measured torques were transformed to the frequency domain and the magnitude and phase of the ratio is plotted. From this data, we estimate the frequency at −3 dB in the frontal plane to be 21.2 Hz. The corresponding phase lag is −329°. (c), (d) Corresponding data for the test performed in the sagittal plane. We estimate the frequency at −3 dB to be 19.4 Hz with phase lag of −309°.

The feed-forward torque bandwidth of the system was estimated using a similar experimental setup. The crank of the hip actuator was connected to a grounded force-torque sensor (Sunrise Instruments M3713D). The thigh frame was also grounded. We set the actuator’s desired torque in the frontal or sagittal plane to a sinusoidal profile with an amplitude of 20 N·m and a frequency that increased from 0 to 30 Hz over a 30 s period. The forces and torques measured by the force-torque sensor were reflected to the joint center [see Fig. 8(a) and (c)]. A close-up shows the tracking performance between 0 and 4 s (approximately 0 to 4 Hz signal). In this section, the root mean square error between the desired and measured signal is 7.2 N·m in the frontal plane [see Fig. 8(a)] and 6.8 N·m in the sagittal plane [see Fig. 8(c)]. The desired and measured torques were also transformed into the frequency domain using a fast-Fourier transform (MathWorks MATLAB). We obtain the magnitude and phase of the ratio of the measured to desired torque [see Fig. 8(b) and (d)]. From this data, we estimate the −3 dB crossing as 21.2 and 19.4 Hz in the frontal and sagittal plane, respectively. The corresponding phase lag at the −3 dB crossing is −329° and −309° in the frontal and sagittal plane, respectively.

**Fig. 9.**
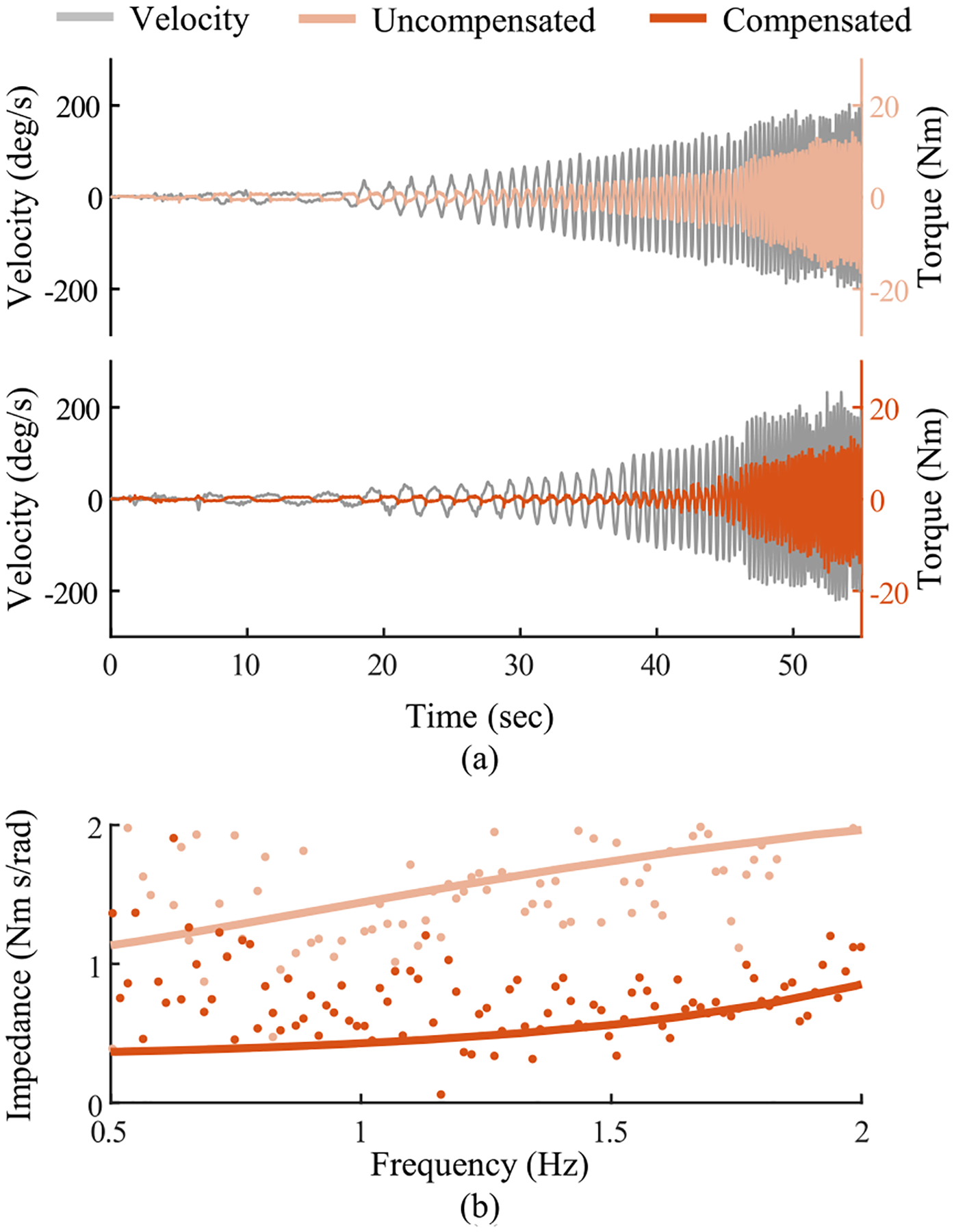
(a) Uncompensated (pink) and compensated (orange) actuator back driving torque and input velocity (gray). (b) Raw output impedance (dots) and estimated impedance (solid line) obtained through fitting a 2-pole and 1-zero transfer function between reflected torque and joint velocity.

The output impedance of the hip actuator was estimated by mounting the crank of the exoskeleton to a grounded six-axis force-torque sensor and manually driving the frame through a sinusoidal motion in the sagittal plane to mimic walking at different frequencies. Forces and torques measured by the load cell were reflected to the joint center. We performed the test under two conditions, with and without feedforward compensations, the results of which are shown in Fig. 9. The output impedance was estimated in the frequency domain by fitting a 2 pole 1 zero transfer function between reflected joint torque and joint velocity. We estimate the reflected damping and inertia without compensations to be 1.13 N·m/rad/s and 0.53 N·m/rad/s^2^. With motor compensations on, the reflected damping and inertia decrease to 0.37 N·m/rad/s and 0.35 N·m/rad/s^2^. The feedforward compensations decrease the reflected damping by 68% and the reflected inertia by 35%. These results suggest that an exoskeleton user will experience limited resistance when walking or running.

## VI. Human Experiments

### A. Methods

The performance of the control algorithm, the torque capability of the device, and the ability of the unilateral hip exoskeleton to modify step width were tested on five healthy young subjects (body mass 75±19kg, age 25±2.5 years, mean ± standard deviation) that were familiar with the exoskeleton operation (see Table IV). The experimental protocol was approved by the University of Utah Institutional Review Board. Written informed consent was provided by the subjects before the experiment took place. The subjects consented to disseminate pictures and videos of the experiment.

Each subject tested five experimental conditions aiming to assess the relationship between frontal plane assistance and step width. The experimental conditions were transparent mode (i.e., no exoskeleton assistance), and sagittal plane assistance with either positive or negative frontal plane assistance during the stance or swing phase of walking, for a total of five different experimental conditions. The order of the experimental conditions per subject was randomized.

At the start of the experiment, the subjects familiarized themselves with the exoskeleton and control algorithm for at most 30 min. During this familiarization period, the subjects walked on an instrumented treadmill (Bertec) at their self-selected walking speed for 1–2 min while an experimenter tuned the exoskeleton assistance timing and duration in the sagittal and frontal plane based on experimenter expertise and user feedback. This process was performed for the five assistance profiles (e.g., one sagittal plane assistance profile and four frontal plane assistance profiles) for a total of less than 10 min of assisted walking. We also used this time to finalize the exoskeleton fit by adjusting the pelvis and thigh interfaces as needed until a comfortable fit was found. Thus, the familiarization period ranged from a few minutes to, at most, 30, depending on the required exoskeleton adjustments.

**Fig. 10.**
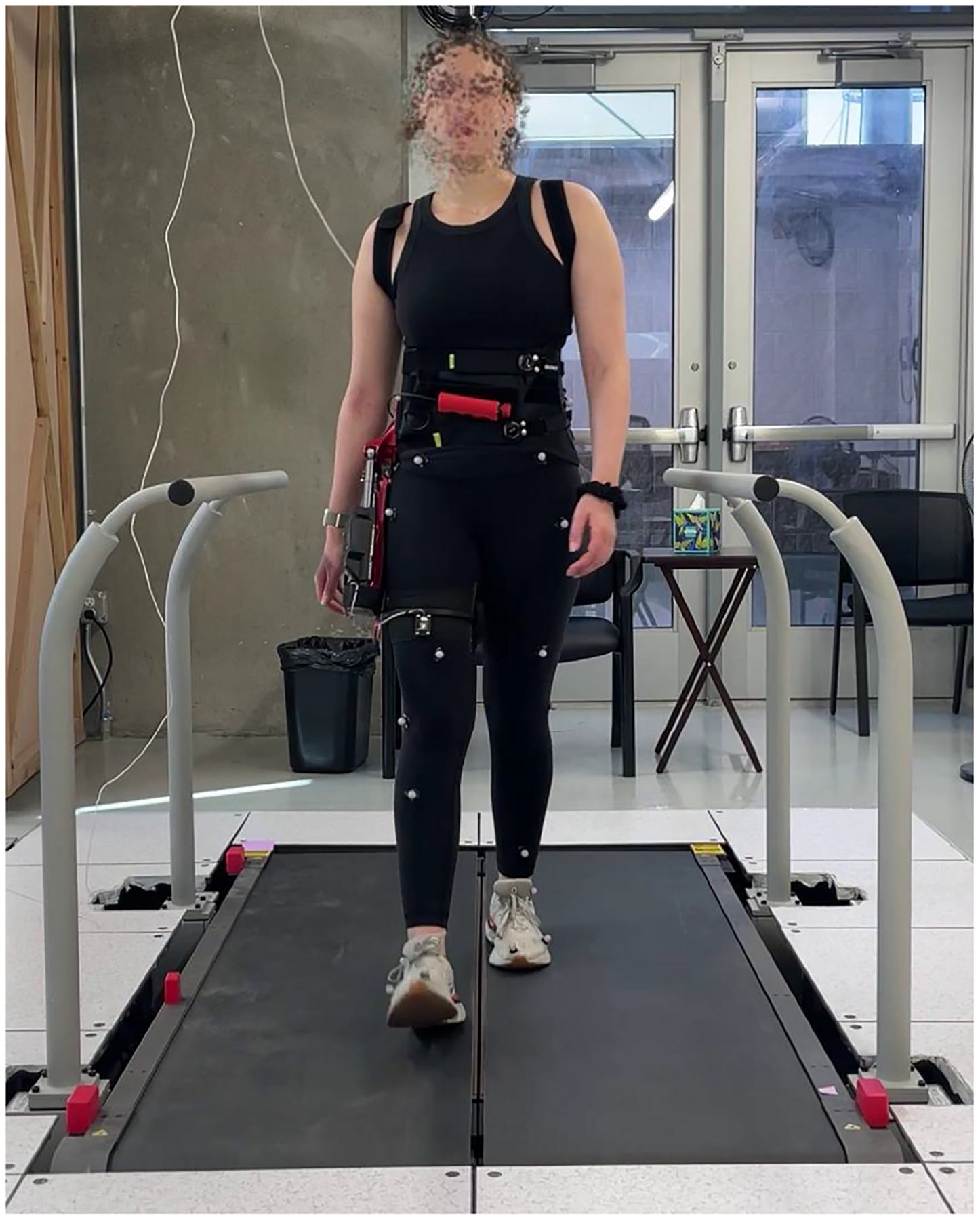
Subject (S1) walked on an instrumented treadmill at their self-selected walking speed with the powered exoskeleton in a unilateral configuration. Reflective marker trajectories were recorded with vicon motion capture cameras.

After familiarization, reflective markers were placed on the lower-limb segments based on a modified Newington-Helen Hayes gait model (Vicon Nexus 2.12) with two additional markers added to each shank and thigh segment to improve marker tracking (Fig. 10, Supplementary Video 1). Then, the subjects walked at their self-selected walking speed for 60 s while receiving one of the assistance conditions. Lower body kinematics and ground reaction forces and torques were recorded using an optical motion capture system and instrumented treadmill (Vicon 3D motion and Bertec). After each assistance condition, the subjects rested for up to two minutes before continuing with the experiment.

**Fig. 11.**
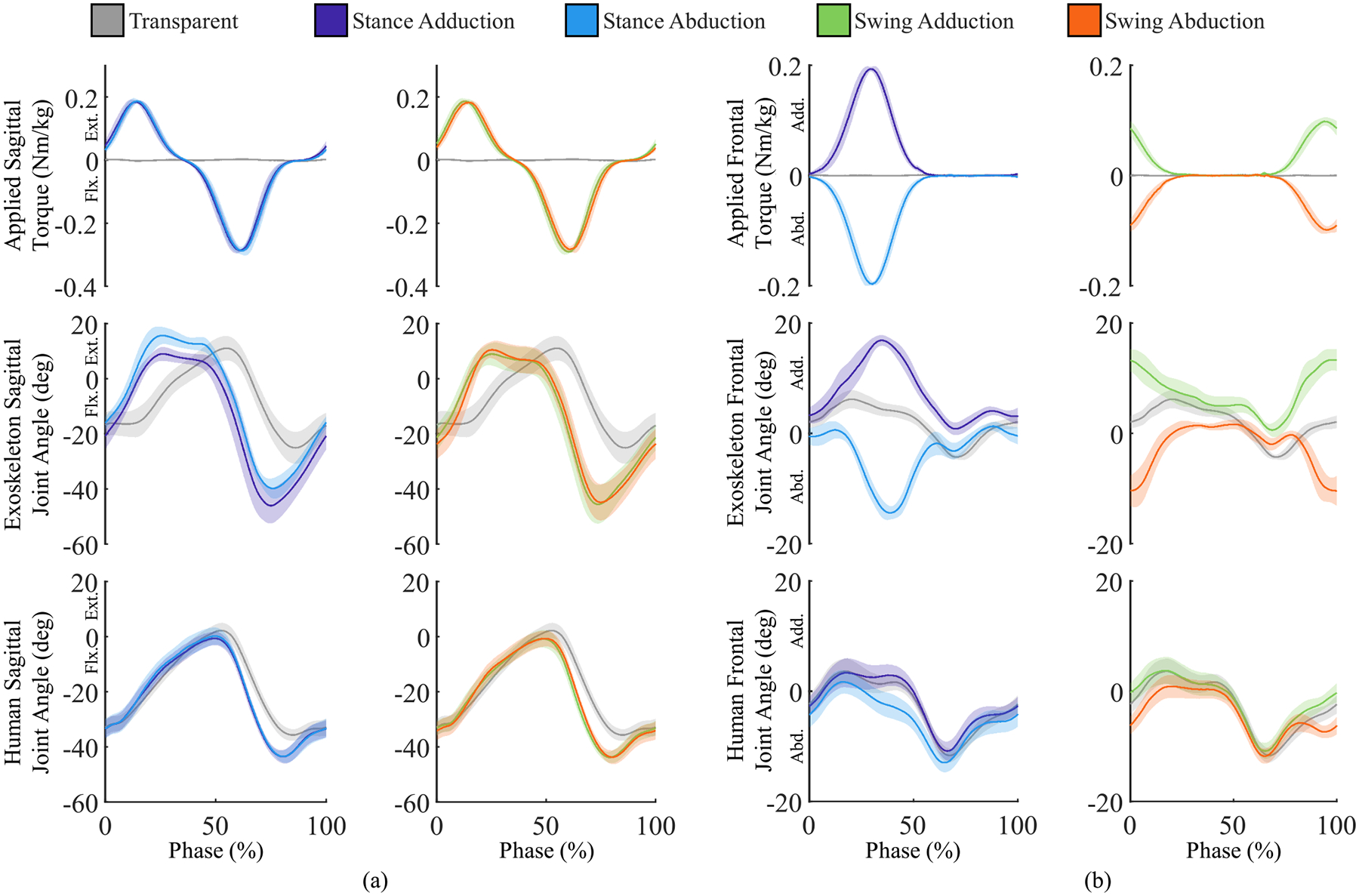
Exoskeleton and human performance for the right actuator and leg across five experimental conditions segmented heel strike to heel strike. Following the convention of Fig. 1, adduction and extension are considered positive. (a) Sagittal plane exoskeleton applied torque, exoskeleton joint angle, and human joint angle. (b) Frontal plane exoskeleton applied torque, exoskeleton joint angle, and human joint angle. The mean (solid line) and standard error of the mean (shaded region) are shown.

In each experimental condition, subjects walked at their self-selected speed on an instrumented treadmill while receiving a Gaussian shaped assistance profile scaled by body weight [see Figs. 10, 11(a) and (b), top plot]. The sagittal plane assistance profile was inspired by our previous research assisting the residual limb of transfemoral amputees [7], [47]. The frontal plane profiles and timing were selected to explore the impact of frontal plane torque on step width and to match prior research that explored abduction assistance during the stance phase of walking [46]. The magnitude of the assistance profile was determined following pilot experiments with the heaviest subject [48]. During these pilot studies, the experimenters tuned the torque profiles to achieve the maximum level of assistance without reaching the voltage limit of the battery or the continuous current limit of the motor. This maximum torque was then normalized by body weight. The flexion assistance magnitude was set to 0.32 N·m/kg and peaked at toe-off, while the extension assistance magnitude was set to 0.21 N·m/kg and peaked immediately after heel strike. Frontal plane assistance during stance was tuned to peak midway through stance phase and set to 0.21 N·m/kg. Frontal plane assistance during swing was tuned to peak immediately before heel strike to have the largest impact on step width and peaked at 0.11 N·m/kg.

For each subject and condition separately, we segmented the exoskeleton assistive torque, joint angle, and estimated gaitphase, as well as the subject’s hip angle and stride width into individual strides using the ground reaction force for the right leg as measured by the instrumented treadmill. After segmentation, we averaged the last ten strides to calculate the mean trajectory for the exoskeleton applied torque and joint angle and for the biomechanical hip angle for each experimental condition and subject. For the last ten strides, we also calculated the maxima and minima of the applied torque, the time of peak sagittal plane torque, the average step width during double support, and the average value of exoskeleton phase reset and averaged them for each subject, experimental condition, and variable. Unless otherwise stated, values are reported as mean ± standard error of the mean.

### B. Results

Across the powered trials, the exoskeleton provided an average peak flexion assistance of 0.32 N·m/kg and an average peak extension assistance of 0.21 N·m/kg [see Fig. 11(a), top plot]. The average peak frontal plane assistance during swing was 0.11 N·m/kg while the average peak frontal plane assistance during stance was 0.21 N·m/kg [see Fig. 11(b), top plot]. For the heaviest subject, the peak sagittal plane torque was 30.3 N·m and the peak frontal plane torque was 20.2 N·m. The peak sagittal plane torque occurred at 61.0±1.7%,62.0±1.7%,60.2±1.7%,61.3±1.7% of gait phase for the four assistance conditions: stance adduction, stance abduction, swing adduction, and swing abduction. Across the four powered conditions and subjects, the exoskeleton hip actuator produced an average of 33.2±1.5 J (range: 24.9 to 55.6 J) of mechanical energy and consumed an average of 40.1±1.6 J (range: 29.3 to 71.8 J) of electrical energy per stride. This equates to an average efficiency of 84.1%±2.5% (range: 74.0% to 92.3%). Considering the 6 W of electric power used by the high-level electronics, the bilateral exoskeleton equipped with a 128 kJ battery can assist on average 3120±80 steps (range: 1700 steps to 3900 steps).

Exoskeleton assistance modified the exoskeleton and human hip joint kinematics. The magnitude of both the exoskeleton and anatomical hip flexion angle at peak flexion assistance increased between the transparent and four powered conditions [see Fig. 11(a)]. In contrasts, only the magnitude of the exoskeleton extension angle at peak extension assistance increased substantially between the transparent mode and the four powered conditions. The magnitude of the exoskeleton and anatomical hip abduction angle at peak stance and swing abduction assistance increased between the transparent mode and the two powered conditions with abduction assistance [see Fig. 11(b), blue and orange lines]. In contrast, only the adduction exoskeleton joint angle at peak stance and swing adduction assistance increased substantially between the transparent mode and the two powered conditions with adduction assistance [see Fig. 11(b), purple and green lines].

**Fig. 12.**
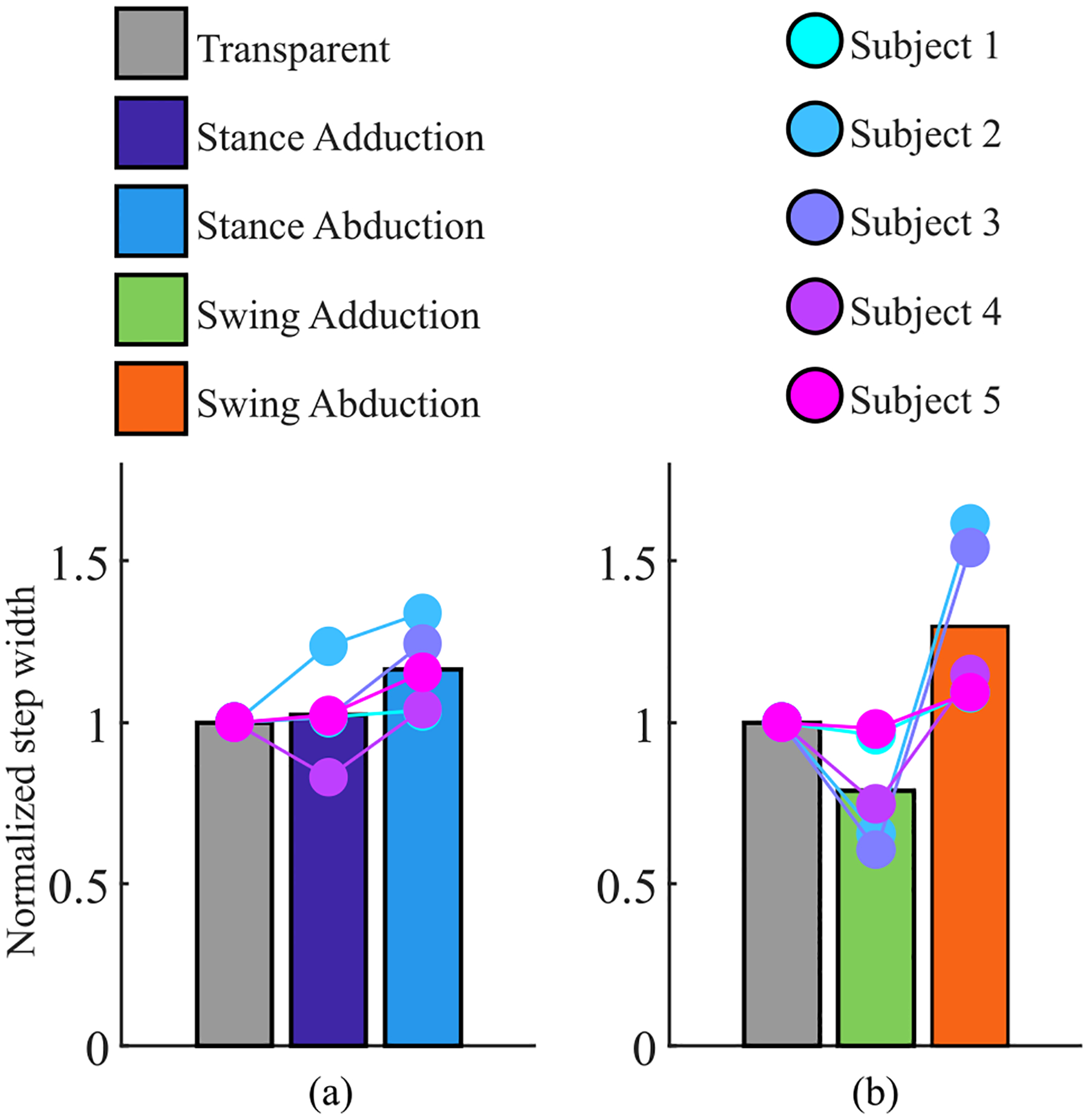
Average normalized step width of the subjects (bars) with respect to transparent mode for frontal plane assistance during (a) the stance phase and (b) the swing phase of walking. Individual subject performance is plotted in dots.

Exoskeleton assistance in the frontal plane modified the base of support. When the exoskeleton applied adduction or abduction torque during stance, the step width normalized by the baseline (transparent mode) was 1.05±0.07 and 1.14±0.04, respectively [see Fig. 12(a)]. In contrast, when the exoskeleton applied adduction or abduction torque during swing, the step width normalized by the baseline was 0.86±0.07 and 1.24±0.08, respectively [see Fig. 12(b)].

Gait phase evolution was estimated by the exoskeleton using an adaptive oscillator and finite-state machine. Across the four powered experimental conditions, the phase estimate reset on average at 101% and the maximum error at phase reset was 3.1%.

## VII. DISCUSSION

Powered hip exoskeletons have the potential to improve gait balance and efficiency in clinical populations by concurrently generating torques in the frontal and sagittal planes. To the best of our knowledge, only two autonomous powered hip exoskeletons can provide assistive torques in both planes concurrently. Unfortunately, these devices are too heavy and bulky for unrestricted use in the real world, especially for clinical populations with limited strength and balance. To address this issue, we present the first powered hip exoskeleton using a parallel kinematic design with two linear actuators located along the user’s thigh. Because the two linear actuators concurrently provide assistance in abduction/adduction and flexion/extension, the proposed exoskeleton is much lighter than previous designs (e.g., 5.3 kg versus 9.2 kg) while being able to provide similar torque (sagittal plane torque 30 N·m versus 34 N·m, frontal plane torque 20 N·m versus 25 N·m) [25], [38] (see Table V). Moreover, due to its unique parallel actuation, the proposed exoskeleton is compact (adding only 3 cm posteriorly, and 8 cm laterally at the hip), which facilitates use in the real world. Human studies with healthy individuals show that the proposed exoskeleton can assist in the sagittal plane while providing frontal plane torques to alter step width, which is an important indicator of balance [49]. Moreover, comparisons to existing exoskeletons suggest that the proposed parallel actuation system satisfies the size, mass, and torque requirements necessary to potentially improve balance and reduce metabolic cost in individuals with lower limb impairments in the real world [7], [43], [46], [50], [51].

Virtually all powered hip exoskeletons that provide assistance in the frontal plane use a *series* kinematic design, and place the frontal-plane actuators posteriorly to the user. However, this posterior location prevents the user from comfortably sitting in a chair [24], [25], [26]. Furthermore, this posterior location means that at high degrees of hip flexion that occur during sit-to-stand transitions or stair ascent, the abduction and adduction torque generate hip internal/external rotation. By using a parallel kinematic design, we can place the actuators laterally to the user’s thigh, which reduces the posterior dimension of the exoskeleton from more than 10 to 3 cm while maintaining a lateral profile of 8 cm which is similar to, if not less than, most bilateral hip exoskeletons (see Table V). Depending on the chair and individual, the low-profile design may allow users to sit comfortably without the exoskeleton contacting the arms or back. Moreover, the frontal plane joint of the proposed hip exoskeleton is located distal to the sagittal plane joint. This kinematic configuration reduces the actuator torques that cause hip internal/external rotation. Thus, the proposed parallel actuator enables the proposed hip exoskeleton to provide both propulsive and stabilizing torques without hindering common activities like sitting, arm swinging, and ascending stairs.

In the proposed *parallel* kinematic design, two linear actuators concurrently generate forces to control the hip abduction/adduction and flexion/extension. In contrast, in a *series* kinematic configuration, each actuator independently generates abduction/adduction or flexion/extension. As a result, the proposed parallel actuator has torque density of 17.6 N·m/kg in the sagittal plane and 11.8 N·m/kg in the frontal plane. In contrast, the largest actuator torque density for a series exoskeleton is 12.4 N·m/kg (see Table V).

The use of the proposed parallel mechanism can also lead to increases in exoskeleton torque density and a decrease in exoskeleton size. Exoskeletons that utilize a series configuration to assist in both the frontal and sagittal planes are typically constructed from posterior and lateral actuators that are connected by load transferring frame [24], [25], [38], [43], [52]. These actuators add 6.5 to 10 cm lateral and 13 to 17 cm posterior to the user. In contrast, the proposed parallel actuator is only located lateral to the user which decreases the posterior dimension to 3 cm while maintaining a lateral profile of 8 cm. Additionally, linkages in the parallel actuator function as structural frames that transfer load directly to the user. Thus, no additional structural parts other than the pelvis and thigh brace are needed to complete the exoskeleton. This results in an exoskeleton torque density of 5.7 N·m/kg in the sagittal plane and 3.8 N·m/kg in the frontal plane which is 41%–53% larger than the next largest exoskeleton torque density (3.7 and 2.7 N·m/kg, respectively [25], [38], see Table V).

The proposed exoskeleton does not use force sensing and closed-loop control, which is similar to our previous powered exoskeletons and prostheses using linear actuators [30], [32], [33], [34]. Bench top testing shows that the feed forward compensations reduce the reflected damping and inertia by 68% and 35%, respectively, achieving high backdrivability. Analysis of step response performance indicates that the hip actuator has a maximum steady state RMS error of 1.0±0.1N·m for 15 N·m torque steps and an RMS error 0.7±0.0N·m for 5 N·m torque steps. This performance is similar to other hip exoskeletons using open- and closed-loop control [30], [43], [53].

Analysis of the chirp performance on the benchtop indicates a −3 dB crossing of 21.2 Hz in the frontal plane and 19.4 Hz in the sagittal plane [see Fig. 8(b) and (d)], which is similar to other exoskeletons [30], [54], [55], [56], [57]. However, there is substantial phase lag (about 300°) at the −3 dB crossing and signal amplification (about 11 dB). Signal amplification and large phase lag in the torque response of wearable robots are not uncommon. Signal amplification can be as large as 4 to 10 dB and occur at frequencies between 6 and 100 Hz. Moreover, phase lag at the −3 dB frequency can be between −100° and −360°, occurring at frequencies of 10–60 Hz [29], [55], [56], [57], [58]. However, these performance characteristics represent the performance of the torque controller as the exoskeleton interacts against an external frame with substantially higher stiffness than the human body, affecting the frequency spectrum of the torque response. Most importantly, the power spectrum calculated from human biomechanics datasets indicates that 99.7% of kinematics occur at frequencies lower than 4.8 Hz [59], which is substantially lower than the resonance frequency and −3 dB bandwidth. Similarly, the power spectrum of the desired torque control used during our human subject experiments indicates that 90% of the signal energy is below 3 Hz. At this frequency, the hip actuator magnitude is about 1.1 to 1.3 dB while the phase lag is about −25° to −30°. This analysis, combined with the low overshoot and RMS error during torque step response, suggests that the proposed exoskeleton satisfies the necessary requirements for human subject testing.

The maximum signal amplification for the mechanism presented here is slightly higher than previously reported values (10.5–11.6 dB compared to 10 dB) [58], while the phase lag is higher than some previously reported values [57], [60]. The observed signal amplification and phase lag may be due to several factors, including test bench setup, backlash in the transmission mechanism, implementation of low-level control, or parallel actuation. However, inferences derived from a comparison of results presented by different authors are limited due to different control strategies (open- versus closed-loop), test conditions (measurement of rotary or linear actuator performance versus assembled exoskeleton), and measurement methods (external load cells versus internal load sensing). Future studies should investigate how different factors affect the frequency response of a powered exoskeleton.

Level ground walking with five human subjects verified that the exoskeleton can produce up to 30 N·m of sagittal plane torque and 20 N·m of frontal plane torque concurrently [see Fig. 11(a) and (b), top plot]. These torque peaks correspond to providing 34% and 20% of the biological sagittal and frontal plane torque of a 95th percentile male [17], [37]. Studies with clinical populations and healthy individuals indicate that these torques are sufficiently large to reduce metabolic consumption in overground walking for a 95th percentile male through applying either frontal or sagittal plane torques [7], [46], [50], [51]. Similarly, studies with healthy individuals indicate that this is sufficient torque to improve stance width and measures of balance in both steady state and transient (as-needed) operations [43], [46]. In agreement with these studies, our human testing shows that abduction frontal plane assistance during swing increased step width from the baseline condition by an average of 24%, whereas adduction frontal plane assistance during swing decreased step width from the baseline condition by an average of 14% [see Fig. 12(b)]. These results show that the proposed exoskeleton can modify stride width and, indirectly, the base of support, suggesting that the proposed exoskeleton may positively impact balance [49].

Frontal plane assistance during swing impacted step width (see Fig. 12). Specifically, frontal plane abduction torques during swing increased step width by 24% [see Fig. 12(b)] while frontal plane adduction torques during swing decreased step width by 14%. A recent study using an autonomous exoskeleton with only powered frontal plane hip assistance demonstrated the ability to increase step width by an estimated 57% and decrease step width by an estimated 31% [26]. These changes in step width are much higher than the ones we observed in this study. However, their exoskeleton applied admittance control to both legs for the entire duration of the gait cycle with a virtual equilibrium position of either ±15° and a stiffness of 60 N·m/rad. In contrast, we applied frontal plane torques to only one leg during either the swing or stance phase of the gait cycle [see Fig. 11(b), top plot]. Specifically, in our experiment, the abduction torque applied in swing lasted for about 40% of the gait cycle and increased step width by 24% [see Fig. 12(b)], which is roughly half of the change in step width observed with constant bilateral admittance control used in prior studies [26]. Similarly, in our study, the adduction torque applied in swing lasted for about 40% of the gait cycle and decreased step width by 14% [see Fig. 12(b)], which is, roughly half the change in step width observed in prior studies [26]. This comparison suggests that assistance magnitude, duration, and control strategy may impact the relative change in step width.

Frontal plane assistance during stance impacted step width [see Fig. 12(a)]. Specifically, abduction and adduction assistance applied to the stance limb increases step width by 14% and 5%, respectively. Similar to prior work [46], all five subjects increased their step width when receiving abduction assistance during stance, suggesting that abduction assistance during stance has the potential to improve balance. In contrast, adduction assistance decreased step width for only one of the five subjects. Moreover, the variation in subject response was large. One subject increased step width by 23%, three subjects had little change in step width, and one decreased step width by 17%. This variation in result may be related to balance. Exoskeleton torques applied during the stance phase may impact both foot placement and the center of mass trajectory. As a result, the center of mass may deviate from its natural course and the subject may take wider steps to increase their base of support. Thus, frontal plane torques applied during stance may positively or negatively influence measures of balance or gait efficiency. Additional biomechanical studies are needed to better understand the relationship between frontal plane torques applied during the stance phase of walking and gait adaptations.

The proposed kinematic model and simulation framework indicate that the exoskeleton hip actuator consumes 24.5 J of electrical energy per stride. In contrast, the electrical energy consumption observed in the subject testing was between 29.3 and 71.8 J per stride. The difference between the simulated and real energy consumption is primarily attributed to the exoskeleton torque timing and increase in exoskeleton hip joint range of motion. Specifically, in the sagittal plane, the experimental torque profile is timed such that extension torques are aligned with extension velocity and flexion torques are aligned with flexion velocity [see Fig. 11(a)]. Similarly, the experimental frontal plane torque caused large deformation of the exoskeleton interfaces in the direction of the applied torque [see Fig. 11(b), top and middle plot]. Thus, during the human subject experiments, the exoskeleton primarily performed positive power (net mechanical energy of 24.9 to 55.6 J per stride) at the cost of 29.3 to 71.8 J of electrical energy. This equates to an average efficiency of about 84%. In contrast, the simulation was based on torque profiles from biomechanical datasets of level walking, which include both positive and negative power phases [17]. The simulation predicts that the exoskeleton will inject about 11 J of mechanical energy while absorbing about 7 J of mechanical energy. In total, accounting for thermal losses, this costs 24.5 J of electrical energy, which equates to an expected efficiency of 15%. Thus, the increase in electrical energy consumption can be largely explained by the increase in net mechanical power produced by the exoskeleton. Moreover, this analysis highlights the large difference between simulation and experimental performance, and indicates that device efficiency can be highly impacted by negative power phases.

Human adaptation to the exoskeleton assistance and motor driver inefficiency may also contribute to an increase in electrical energy consumption of the hip actuator. Results from the human subject experiments [see Fig. 11(a), bottom plot] indicate that, on average, the human hip joint exhibited a greater range of motion in the sagittal plane when assisted by the exoskeleton. Fig. 11 also shows subtle changes in the frontal plane human hip joint angles. In contrast, the simulation assumed the actuator followed human biomechanics data sets which did not account for human adaptation to the exoskeleton assistance. Furthermore, the simulation framework assumes 100% efficiency of the 4-Q servo drives during both positive and negative power phases. However, small inefficiencies may have contributed to increased energy consumption. Further refinement of the simulation framework can be done to better predict exoskeleton actuator energy consumption.

We use an adaptive oscillator and finite state machine to provide a continuous estimate of gait-phase evolution. This control approach has been previously applied to generate sagittal plane assistance in walking, running, and stair ascent [30]. Here we show, for the first time, that this control approach can be used to reliably control frontal plane assistance across different walking speeds (1.5 to 2.4 mph) and assistance conditions (abduction or adduction assistance timed during stance or swing). The timing of assistive torques across the experimental conditions and subject-specific walking speeds was consistent (average timing of peak flexion torque of 61.1±0.3% gait phase across powered conditions), indicating that the adaptive frequency oscillator and finite state machine are robust to differences in assistance profile and walking speed. This result suggests that adaptive oscillators with finite state machines can be used to develop controllers aiming to improve balance.

### A. Limitations

Our study demonstrates the ability of the proposed exoskeleton to produce sagittal and frontal plane torques that have been shown to reduce metabolic consumption and increase measures of balance in steady-state and dynamic conditions [7], [10], [43], [46]. However, these outcomes should be directly studied with this device to understand the impact of both frontal and sagittal plane torques applied continuously or as needed on the metabolic cost of transport and balance of clinical populations. While our research explores the continuous application of Gaussian shaped torque profiles, alternative torque profiles applied continuously or intermittently may elicit a larger impact on step width, balance, or metabolic consumption.

The bilateral exoskeleton developed in this study weighs 5.3 kg and adds 3 cm posterior and 8 cm lateral to a user. While the weight and size of the device is substantially less than previous exoskeletons that provide both frontal and sagittal plane assistance, it may still be too heavy for individuals with lower-limb impairments to use in the real world. Moreover, the size and structure of the interfaces may still be too bulky to accommodate real-world ambulation. For individuals with hemiparesis, it may be possible to reduce the exoskeleton weight by using only one actuator to assist the impaired leg. Studies with clinical populations are necessary to assess the ability of individuals to tolerate the exoskeleton weight and size.

During powered exoskeleton conditions, the exoskeleton range of motion and maximum angle increased substantially (see Fig. 11). While this is primarily due to the application of assistive torque, it has the unintended consequence of increasing the exoskeleton joint range of motion and speed. As a result, the measured motor voltage, joint angle, and energy consumption were considerably higher than expected from simulations. The simulation framework should be improved to include the flexibility of the human–robot interface to provide more realistic results [53]. Alternatively, better orthotic interfaces can be developed to limit deformations.

## VIII. Conclusion

Powered hip exoskeletons that generate torque in both the sagittal and frontal planes are fundamental to improving gait economy and balance in individuals with poor mobility. However, existing technologies are too bulky and heavy to use in the real world. This article contributes the kinematic model, mechatronic design, and benchtop and human subject testing of a hip exoskeleton with a parallel actuator. This parallel actuator enables a powered hip exoskeleton that is substantially lighter, more compact, and more ergonomic than previous devices, while still generating similar levels of torque. Human studies with five healthy young adults show that the proposed powered hip exoskeleton, controlled with a state machine and adaptive frequency oscillators, can consistently generate torques in the frontal and sagittal plane. Moreover, the application of torques in the frontal plane alters step width, a key component of balance. Future work will explore control strategies that intentionally alter step width to improve balance in clinical populations.

## Supplementary Material

supp1-3539172

## Figures and Tables

**TABLE I:** Design Parameters

Parameter	Unit	Value
$V_{s}$	V	29.6
$k_{t}$	mNm/A	14
$R_{m}$	Ω	0.527
$I_{\text{nom}}$	A	4.64
$H_{m}$	${\text{gcm}}^{2}$	7.68
$H_{\text{TR}}$	${\text{gcm}}^{2}$	49.9
$\eta_{\text{TR}}$		0.9
$\eta_{\text{driver}}$		0.9
${\text{TR}}_{g}$	rad/rad	3.0
${\text{TR}}_{bs}$	rad/m	2513
$v_{11}^{0}=\left(x_{11},y_{11},z_{11}\right)$	mm	(−26, −30, −23)
$v_{13}^{0}=\left(x_{12},y_{12},z_{12}\right)$	mm	(−3, −245, −30)
$v_{21}^{0}=\left(x_{21},y_{21},z_{21}\right)$	mm	(−25, 7, 37)
$v_{23}^{0}=\left(x_{22},y_{22},z_{22}\right)$	mm	(−3, −245, 46)
$v_{12}^{0}=\left(x_{2},y_{2},z_{2}\right)$	mm	(0, −17, 0)

**TABLE II:** Exoskeleton Weight Breakdown

System	Mass (g)	
Linear Actuator, Motor Driver	406 × 2	*Hip Actuator* *1717 × 2* *64.8% of total mass*
Frame and Crank	736	
Low Level Electronics Boards	109	
Protective Covers	60	
Thigh Passive DOF	38 × 2	*Orthoses, Braces* *1298 × 1* *24.5% of total mass*
Thigh Orthosis	140 × 2	
Pelvis Orthosis	942 × 1	
High Level Electronics boards	30	*High Level* *Electronics* *603 × 1* *11.4% of total mass*
Battery	220	
Power and Data Cables	96 × 2	
Case and Fans	161	
Bilateral Exoskeleton	5.3 kg	

**TABLE III:** Step Response Results

	Torque Step (N·m)		Risetime (ms) (mean ± std. dev)		Percent Overshoot (%) (mean ± std. dev)		RMS Steady State Error (N·m) (mean ± std. dev)	
	Frontal plane	Sagittal plane	Frontal plane	Sagittal plane	Frontal plane	Sagittal plane	Frontal plane	Sagittal plane
Frontal	5.0	0.0	14.4±1.3		8.9±0.8		0.2±0.0	0.7±0.0
Plane Step	15.0	0.0	16.6±0.4		16.3±0.6		1.0±0.1	0.2±0.1
Sagittal	0.0	5.0		9.4±0.5		13.9±1.0	0.3±0.1	0.2±0.1
Plane Step	0.0	15.0		11.0±1.2		18.4±1.6	0.3±0.1	0.3±0.3
Combined	5.0	5.0	17.2±1.1	16.8±1.1	6.9±0.4	8.7±0.6	0.2±0.0	0.4±0.1
Step	15.0	15.0	16.0±1.0	16.0±1.4	31.0±1.0	39.3±2.0	0.5±0.2	0.8±0.1
Average			16.1±1.2	13.3±3.7	15.8±10.9	20.1±13.4	0.4±0.3	0.4±0.3

**TABLE IV:** Subject Demographics

	S1	S2	S3	S4	S5
Gender	F	M	F	M	M
Age (yr)	25	29	22	25	24
Walking Speed (mph)	2.0	2.4	2.4	2.4	1.5
Mass (kg)	69.1	63.6	54.0	96.4	95.0

**TABLE V:** Comparison of Autonomous Exoskeletons That Generate Frontal and Sagittal Plane Hip Torques

	Actuator Weight per Hip (kg)	Total Weight (kg)	Lateral Dimension per Actuator (cm)	Posterior Dimension (cm)	Measured Torque		Actuator Torque Density		Exoskeleton Torque Density	
					Sagittal (N·m)	Frontal (N·m)	Sagittal (N·m/kg)	Frontal (N·m/kg)	Sagittal (N·m/kg)	Frontal (N·m/kg)
**Utah (this study)**	**1.7 × 1**	**5.3**	**8**	**3**	**30.3**	**20.2**	**17.6**	**11.8**	**5.7**	**3.8**
Mindwalker* [24], [61], [62], [63]	2.9 × 2	28	>9^+^	15^+^	31	50	5.3	8.6	1.1	1.8
NCSU [25], [38]	1.5 × 2	9.2	9^+^	17^+^	34	25	11.3	8.3	3.7	2.7
Panasonic [64]	0.58 × 4	9.3	10^+^	>13^+^	10	10	4.3	4.3	1.1	1.1
Georgia Tech [52]	0.485 × 2	7.76	6.5	13^+^	12	12	12.4	12.4	1.5	1.5

Note:

* Hip, Knee, ankle exoskeleton,

+ estimation of the dimension.
